# Hesperetin promotes longevity and delays aging via activation of Cisd2 in naturally aged mice

**DOI:** 10.1186/s12929-022-00838-7

**Published:** 2022-07-24

**Authors:** Chi-Hsiao Yeh, Zhao-Qing Shen, Tai-Wen Wang, Cheng-Heng Kao, Yuan-Chi Teng, Teng-Kuang Yeh, Chung-Kuang Lu, Ting-Fen Tsai

**Affiliations:** 1 Department of Thoracic and Cardiovascular Surgery, Chang Gung Memorial Hospital, Linkou, Taoyuan, Taiwan; 2 College of Medicine, Chang Gung University, Taoyuan, Taiwan; 3 Community Medicine Research Center, Chang Gung Memorial Hospital, Keelung, Taiwan; 4 Department of Life Sciences and Institute of Genome Sciences, National Yang Ming Chiao Tung University, 155 Li-Nong St., Sec. 2, Peitou, Taipei, 11221 Taiwan; 5 Center of General Education, Chang Gung University, Taoyuan, Taiwan; 6 Institute of Biotechnology and Pharmaceutical Research, National Health Research Institutes, Miaoli, Taiwan; 7 National Research Institute of Chinese Medicine, Taipei, Taiwan; 8 Institute of Molecular and Genomic Medicine, National Health Research Institutes, Miaoli, Taiwan; 9 Center for Healthy Longevity and Aging Sciences, National Yang Ming Chiao Tung University, Taipei, Taiwan

**Keywords:** Longevity, Natural aging, Cisd2, Hesperetin

## Abstract

**Background:**

The human CISD2 gene is located within a longevity region mapped on chromosome 4q. In mice, Cisd2 levels decrease during natural aging and genetic studies have shown that a high level of Cisd2 prolongs mouse lifespan and healthspan. Here, we evaluate the feasibility of using a Cisd2 activator as an effective way of delaying aging.

**Methods:**

Hesperetin was identified as a promising Cisd2 activator by herb compound library screening. Hesperetin has no detectable toxicity based on in vitro and in vivo models. Naturally aged mice fed dietary hesperetin were used to investigate the effect of this Cisd2 activator on lifespan prolongation and the amelioration of age-related structural defects and functional decline. Tissue-specific Cisd2 knockout mice were used to study the Cisd2-dependent anti-aging effects of hesperetin. RNA sequencing was used to explore the biological effects of hesperetin on aging.

**Results:**

Three discoveries are pinpointed. Firstly, hesperetin, a promising Cisd2 activator, when orally administered late in life, enhances Cisd2 expression and prolongs healthspan in old mice. Secondly, hesperetin functions mainly in a Cisd2-dependent manner to ameliorate age-related metabolic decline, body composition changes, glucose dysregulation, and organ senescence. Finally, a youthful transcriptome pattern is regained after hesperetin treatment during old age.

**Conclusions:**

Our findings indicate that a Cisd2 activator, hesperetin, represents a promising and broadly effective translational approach to slowing down aging and promoting longevity via the activation of Cisd2.

**Supplementary Information:**

The online version contains supplementary material available at 10.1186/s12929-022-00838-7.

## Background

*Aging is a global burden*. The demographic transition to an aging population is occurring globally via longer life expectancy [1]. Aging is the accumulation of lifelong molecular and cellular damage that result in progressive co-morbidities that affect multiple organ systems together with the co-occurrence of multiple age-associated diseases, these increase mortality and morbidity [2]. Currently very few pharmacological treatment options are available to meet the goal of extending a healthy lifespan while concurrently reducing the likelihood of disability before death [3].

*Mitochondrial dysfunction is a remarkable hallmark of aging*. Many cellular and molecular hallmarks of aging had been identified and categorized [4]. Specifically nine candidate hallmarks have been established, namely DNA damage, telomere loss, epigenetic alterations, loss of proteostasis, dysregulation of nutrient sensing, mitochondrial dysfunction, cellular senescence, stem cell exhaustion, and alterations to intercellular communication. It is likely that there are extensive interconnections between these hallmarks of aging. Accordingly, the experimental amelioration of one particular hallmark is likely to impinge on the others, and all together they will determine the aging phenotype.

Although the molecular pathways contributing to aging are not fully understood, mitochondrial dysfunction does play a crucial role in driving age-associated pathophysiology [5]. Mitochondrial dysfunction is able to accelerate aging in mammals [6–8]. Previous studies have revealed that preservation of mitochondrial function is a key player in maintaining the integrity of many organ systems [9]. In addition, mitochondrial dysfunction, together with perturbation to metabolic flexibility [10], result in a decrease in oxygen consumption and lower ATP production; these have been observed in older subjects who have low physical performance [11] and are a mortality risk factor [12]. Furthermore, mitochondrial dysfunction during aging alters mitochondrial energy metabolism, which, in turn, disturbs the nutrient sensing pathways that regulate glucose homeostasis and insulin sensitivity. These impaired cellular responses compromise whole body energy metabolism and ultimately increase the risks of age-related disease [10].

*Cisd2 is one of the prolongevity genes that mediate lifespan in mammals*. Genetically modified mouse models have revealed prolongevity genes and led to the discovery of various related pathways that modulate mammalian lifespan [13, 14]. According to the databases collected by the Human Aging Genomic Resources, currently there are eight genes (Bub1b, Cisd2, Klotho, Pawr, Pparg, Pten, Sirt1, and Sirt6) that have been experimentally proved to effectively decrease and increase lifespan by genetic knockout and overexpression in mice [15]. Another report from Pedro de Magalhães et al [14] used the Gompertz mortality rate model [16] to determine whether previously reported aging-related mouse genes statistically affect the demographic rate of aging. Interestingly, out of the 30 genes reported to potentially extend lifespan in mice, only two genes, namely Cisd2 [17] and hMTH1 [18], were found to have evidence linking them to slowing aging.

In humans the CISD2 gene (CDGSH Iron Sulfur Domain 2; synonyms ERIS, Miner1, NAF-1, WFS2, and ZCD2) is mapped to chromosome 4q22-24 and is located within a region where a genetic component for human longevity has been reported [19]. Although the interval on chromosome 4q22-25 has only modest evidence associating it with human longevity [20], nevertheless, in mice, we have demonstrated that Cisd2 deficiency shortens lifespan via premature aging [6]. Notably, the level of Cisd2 mRNA and protein decreases during mouse normal aging [21]. Furthermore, we have shown that a persistently high level of Cisd2 in mice, achieved by transgenic expression, extends their median and maximum lifespan without any apparent deleterious side effects. Moreover, Cisd2 protects mitochondria from age-associated damage and functional decline, as well as attenuating age-associated reduction in whole-body energy metabolism [17]. These results suggest that Cisd2 is a fundamentally important regulator of lifespan, and they provide an experimental basis for exploring the candidacy of CISD2 in human longevity.

*Cisd2 maintains Ca*^*2+*^* homeostasis and mitochondrial function in order to mediate lifespan and healthspan.* Our previous studies have revealed that Cisd2 is essential to maintaining intracellular Ca^2+^ homeostasis and mitochondrial integrity in multiple organ systems [22–24]. The Cisd2 protein is located on the endoplasmic reticulum membrane and outer membrane of mitochondria; these locations may provide a means of endoplasmic reticulum-mitochondrial crosstalk through electron transfer in response to redox stimuli [25]. Direct interaction between endoplasmic reticulum and mitochondria is crucial for Ca^2+^ transfer and cellular function [26, 27]. In fact, there is increasing evidence linking altered communication between the endoplasmic reticulum and mitochondria to apoptosis [28], metabolic diseases [29], and aging [26]. As a proof-of-principle genetic approach, we have provided evidence demonstrating that in the heart, Cisd2 deficiency disrupts Ca^2+^ homeostasis via dysregulation of Serca2a activity, which results in Ca^2+^ dysregulation, mitochondrial Ca^2+^ overload and dysfunction, thereby impairing cardiac function. Most strikingly, in Cisd2 transgenic mice, increased Cisd2 expression delays cardiac aging and ameliorates age-related cardiac dysfunction [24, 30]. Consistently, an elevated level of Cisd2 has been found to delay skeletal muscle aging [17], to slow down liver aging [31] and attenuate liver carcinogenesis [23], as well as reducing Alzheimer’s-related neuronal loss [32, 33]. Collectively, these mouse genetic studies confirm that Cisd2 is a key player that controls lifespan and healthspan.

Whether Cisd2 can be targeted pharmaceutically and is able to be activated by small compounds remains unclear. In this study, our aim is to evaluate the feasibility of using a Cisd2 activator as an effective regimen for delaying aging. We anticipate that the pharmaceutical activation of Cisd2, whose expression otherwise decreases during the natural aging of mice, will have therapeutic benefits in terms of ameliorating age-related functional decline and structural damage. Such a compound may help to slow down aging or even rejuvenate aged tissues in naturally aged mammals, thereby promoting longevity and extending a healthy lifespan.

## Methods

### Generation of the HEK293-CISD2 reporter cell line

A CISD2 bacterial artificial chromosome (BAC) reporter clone was constructed from a 102-kb human BAC clone (CTD-2303J4, Invitrogen, San Diego, CA, USA, #96012) that contained the intact gene of human CISD2 in its native chromosomal setting, together with its flanking upstream and downstream regions. This human BAC clone carries the entire 23.8-kb genomic sequence of the CISD2 coding region, a 31.3-kb upstream region and a 46.9-kb downstream region (Additional file 1: Fig. S1A). To generate the CISD2 BAC reporter clone, IRES-Luc-pA, namely an internal ribosome entry site (IRES), luciferase (Luc) and a polyA signal (pA), was inserted into exon 2 of the CISD2 gene (Additional file 1: Fig. S1B) by in vivo recombineering-based method in *Escherichia coli* [34]. To establish the HEK293-CISD2 reporter cell line, the linearized CISD2 BAC reporter construct and pCI-neo plasmid (Promega, Mannheim, Germany, #E1841) were co-electroporated (250 V with capacitance 500 μF) into the HEK293 cells and selected with 1 μg/mL puromycin (Invitrogen, #A11138-03) for 12–14 days in growth medium (DMEM; Gibco, Carlsbad, CA, USA, #11965) supplemented with 10% fetal bovine serum, 1% glutamine/penicillin/streptomycin, 1% non-essential amino acid, and 1 mM sodium pyruvate. The correct clones for the HEK293-CISD2 reporter system were confirmed by PCR and luciferase reporter assay.

### Luciferase reporter assay

The HEK293-CISD2 BAC reporter cells were seeded on a 96-well plate at 2 × 10^4^ cells/well. After 24 h of seeding, the HEK293-CISD2 BAC reporter cells were treated with different doses of hesperetin (and various other compounds individually) for 24 h. After treatment with each compound, luciferase activity was assayed using a ONE-GloTM Luciferase Assay System kit (Promega, #E6120) following the manufacturer’s instructions. The intensity of luminescence was monitored using an Infinite 200 Microplate Reader (Tecan Group Ltd., Männedorf, Switzerland).

### The noble herb compound library

The herb compound library, which contains 780 samples, was established to systematically screen for CISD2 activators from the 60 noble herbs (Additional file 1: Table S1) that were described in a traditional Chinese medicine book, The Divine Husbandman’s Herbal Foundation Canon (神農本草經, Shén Nóng Běn Cǎo Jīng, written between 200 and 250CE) [35]. Briefly, 50 g of each herb was crushed and extracted with 200 mL of 60% ethanol shaking at 25 °C, this was repeated three times. The extracted solution (600 mL) was partitioned using CH_2_Cl_2_ (600 mL), and then dried on a rotary evaporator. The CH_2_Cl_2_ layer and the 60% ethanol layer were further separated into five fractions using a silica gel-based high performance liquid chromatography (HPLC) column or a C18 HPLC column, respectively. The silica gel column was eluted using 100 mL of 100% CH_2_Cl_2_, CH_2_Cl_2_/methanol (95/5), CH_2_Cl_2_/methanol (9/1), CH_2_Cl_2_/methanol (8/2) and 100% methanol in series to give the C1–C5 fractions. The C18 column was eluted using 200 mL of ddH_2_O, 30% methanol (aq), 60% methanol (aq), 90% methanol (aq), and 100% methanol, in series, to give the M1–M5 fractions. Thus, for each herb there are 13 fractions including the crude extract, the CH_2_Cl_2_ layer, the 60% ethanol layer, C1–C5 fractions, and M1–M5 fractions. Each fraction was dried using a rotary evaporator and stored at − 20 °C.

### Identification of Cisd2 activators

Initially, sophoricoside and genistein from *Sophora japonica* (Additional file 1: Table S1) were identified as Cisd2 activators. Five fractions (CH_2_Cl_2_ layer, C2, C3, C5 and M3) from *Sophora japonica* gave Z-scores > 2; and pure sophoricoside and genistein were obtained from the precipitate of the CH_2_Cl_2_ layer of *Sophora japonica* via a bioassay-guided purification. The precipitate of the CH_2_Cl_2_ layer was dissolved in methanol and purified by semi-preparative reverse phase HPLC (Cosmosil C18 ARII, 10 × 250 mm) under the following conditions: isocratic running 24% acetonitrile/H_2_O for 20 min, then to 60% acetonitrile/H_2_O in 5 min, and hold for 5 min; flow rate at 4 mL/min; monitoring at 200 to 400 nm range of a diode array detector. The structures of sophoricoside and genistein were confirmed by NMR [36, 37] and MS/MS data. Subsequently, based on the chemical structures of sophricoside and genistein, seven structural analogs of these flavonoids, namely baicalin, formononetin, Kaemferol-3-*O*-rhamnoside, medicarpin, puerarin, rutin, and hesperetin were selected and examined for their ability to enhance CISD2 expression using the HEK293-CISD2 reporter cell assay (Additional file 1: Fig. S1C, D). Finally, hesperetin (a single compound with > 98% purity) was identified as a promising Cisd2 activator that is able to enhance Cisd2 expression both in vitro and in vivo (Additional file 1: Fig. S1E–G).

### Analysis of hesperetin and its conjugated metabolites

The levels of hesperetin, hesperetin-7-*O*-beta-d-glucuronide (H7G) and hesperetin-7-*O*-sulfate (H7S) in the serum and tissues of the mice were quantified by LC–MS/MS. The mice were fed the dietary hesperetin supplemented food ad libitum for 4 months until the day of sacrifice. To synchronize the food intake, the mice were fasted for 6 h (2 p.m. to 8 p.m.) and then fed the hesperetin supplemented food for 2 h (8 p.m. to 10 p.m.). Sample preparation for the LC–MS/MS assay consisted of 50 µL of mouse serum or tissue homogenates (liver, cardiac muscle or skeletal muscles) being mixed with 50 µL of 250 ng/mL of hesperetin-d3 (TORONTO Research Chemicals INC., Toronto, Canada, #H289502). Hesperetin-d3 is an internal standard of labelled hesperetin in which the three hydrogens were replaced by deuterium. The mixture was vortexed and then centrifuged at 15,000×*g* for 20 min in a Beckman Coulter Microfuge 22R Centrifuge at room temperature. The supernatant was transferred to a clean tube and finally 15 µL of the supernatant was injected onto the LC–MS/MS system.

### LC–MS/MS analysis

The chromatographic system consisted of an Agilent 1200 series LC system and an Agilent ZORBAX Eclipse XDB-C8 column (5 µm, 3.0 × 150 mm) interfaced with a MDS Sciex API4000 tandem mass spectrometer. The MS/MS ion transitions monitored were m/z 300/9/163.9, 477.0/301.0, 380.9/301.0 and 303.9/163.8 for hesperetin, H7G, H7S and hesperetin-d3, respectively. A gradient HPLC method was employed for separation. The mobile phase A consisted of 10 mM ammonium acetate aqueous solution containing 0.1% formic acid, while the mobile phase B consisted of acetonitrile. The gradient profile was as follows (min/%B): 0.0–0.5/10, 0.5–1.2/60, 1.2–3.4/80 and 3.5–5.0/10. The flow rate was set at 1.5 mL/min into the mass spectrometer with the remainder being split off to waste. The retention times of hesperetin, H7G, H7S, and hesperetin-d3 were 2.46, 2.07, 2.16, and 2.44 min, respectively.

### Mice and hesperetin treatment

The CISD2 reporter transgenic (TG) mice were generated as previously described [38]. Briefly, the linearized CISD2 BAC reporter construct, which carries luciferase as the reporter and driven by the human CISD2 promoter, was microinjected into the pronuclei of fertilized eggs obtained from C57BL/6 mice. The Cisd2 mcKO mice, which carry a Cisd2 KO background specifically in the skeletal and cardiac muscles, were generated as previously described [22]. Briefly, mice carrying the Cisd2 floxed allele (Cisd2 f/f) were bred with transgenic mice carrying the muscle creatine kinase-Cre (MCK-Cre; JAX006475). After two generations of breeding, Cisd2 mcKO (Cisd2f/f;MCK-Cre) mice were obtained. All the mice used in this study are males. All mice have a pure or congenic C57BL/6 background and were housed in a specific pathogen-free facility with a 12–12 h light–dark cycle at constant temperature (20–22 °C). For the dietary hesperetin treatment, old wild-type (WT) mice (19.5 mo to 23.5 mo of age) were provided with a diet (AIN-93G Growth Purified Diet, TestDiet, St. Louis, MO, USA; Additional file 1: Table S2) containing the vehicle (Veh) (3.04% propylene glycol [w/w]; Sigma-Aldrich, Munich, Germany, 16033) with or without hesperetin (0.07% [w/w]; Sigma-Aldrich H4125; purity (HPLC area %) > 95%; 100 mg/kg/day) for 3 to 6 months. After these treatments, the mice were sacrificed using carbon dioxide (CO_2_) inhalation as the method of euthanasia. All animal protocols were approved by the Institutional Animal Care and Use Committee of Chang Gung Memorial Hospital (No. 2017103002 and 2017030901) and National Yang Ming Chiao Tung University (No. 1040104r). The animal protocol was designed to respect the associated guidelines and the 3R principles (Replacement, Reduction and Refinement) according to the “Animal Protection Act” of Taiwan.

### In vivo imaging system (IVIS) analysis

For the in vivo luciferase assay, the luciferase activity in CISD2 reporter TG mice was measured before and after dietary hesperetin treatment (100 mg/kg/day provided in food) using an In Vivo Bioluminescence Imaging System (IVIS) (IVIS 50 System, Xenogen Corp., Alameda, CA, USA)**.** The CISD2 reporter TG mice were injected intraperitoneally with the substrate d-luciferin (150 mg/kg in PBS) and then anesthetized using 2.5% isoflurane in IVIS 50 System for image acquisition. The luminescent intensity at the mouse ventral view was analyzed by living image software 3.2 (IVIS 50 Imaging System, Xenogen Corp.). The bioluminescent signal is presented as mean photons/second/centimeter^2^/steradian (photon/s/cm^2^/sr).

### Serum biochemical and complete blood count (CBC) analyses

Whole blood samples were collected from the facial vein or by cardiac puncture at sacrifice. Serum alanine aminotransferase, aspartate aminotransferase, blood urea nitrogen, creatinine, creatine kinase-MB, total cholesterol, triglyceride, Ca^2+^, Mg^2+^, Na^+^, K^+^, Cl^−^ levels were monitored by Fuji Dri-Chem 4000i (Fujifilm, Tokyo, Japan). Whole blood samples were collected from the facial vein using an EDTA (final concentration 5 mM) coated tube. The CBC was analyzed using a hematology analyzer (model ProCyte Dx, IDEXX, Columbus, OH, USA).

### Whole body composition analysis

Mouse body lean and fat volumes were measured using a micro-CT scanner (SkyScan 1076, Bruker, Kontich, Belgium). The quantitative results for lean, fat, and visceral fat in the whole body of the mice were analyzed using the three-dimensional structure obtained from the micro-CT and software SkyScan 1076 (Bruker).

### Whole-body metabolic rate

A TSE Calorimetry Module of the LabMaster System (TSE Systems GmbH, Homburg, Germany) was used to monitor the oxygen consumption rate (VO_2_), carbon dioxide production rate (VCO_2_), and energy expenditure (EE) of mice. Individual mice were acclimated for 72 h and then assayed for another 48 h using a 12–12 h light-dark cycle (lights on at 8:00 a.m.) with ad libitum access to food and water. The whole-body metabolic rate of each mouse was measured by indirect calorimetry and then corrected according to lean mass, which was calculated as follows: lean mass = lean volume × 1.06 g/cm^3^ muscle density [39].

### Oral glucose tolerance test and insulin tolerance test

For the oral glucose tolerance test, the mice were orally administrated with glucose water (1.5 mg/g) after a 6 h fasting (9:00 a.m. to 15:00 p.m.). Blood samples were collected at the indicated time points [6]. The blood glucose levels were measured using OneTouch Ultra glucose test strips and a SureStep Brand Meter (LifeScan, Milpitas, CA, USA). Serum insulin levels were determined by a mouse insulin ELISA kit (Mercodia, Uppsala, Sweden, #10-1249-01). For the insulin tolerance test, the mice were examined after a 2 h fasting (9:00 a.m. to 11:00 a.m.) and an intraperitoneal injection of insulin (0.75 U/kg) (Actrapid human regular insulin, Novo Nordisk, Bagsværd, Denmark).

### Rotarod trials

Rotarod trials were used to examine the motor coordination, balance and exhaustion resistance of the mice and were conducted using a Rotarod instrument (RT-01, Singa Technology Corporation, Taipei, Taiwan). The mice were placed on a rotarod running at different speeds for the same duration (5 min). Mice were pre-trained three times (5 rpm for 5 min) before the tests. In the test phase, the rotating speed was set at 10, 20 and 30 rpm (the speed up rate was 1 rpm/s). The time of falling was automatically recorded by an infrared sensor at the bottom of the instrument [40].

### Transthoracic echocardiography

Cardiac functions were assessed using a VisualSonics VeVo 2100 Imaging System (VisualSonics, Toronto, Ontario, Canada). Male mice were anesthetized with 1% isoflurane in 95% O_2_. Body temperature was maintained and monitored at 36 °C to 37 °C on a heated pad (TC-1000, CWE Inc., Ardmore, PA, USA). Cardiac function was assessed using a high-frequency 30–50 MHz probe, as described previously [41]. Data analysis was carried out using VisualSonics software. The personnel responsible for data acquisition were blinded to the animal groupings.

### Electrocardiography (ECG)

Functional testing of the mice’s hearts using ECG was performed as described previously [24]. The mice were maintained on a 12:12 h dark–light cycle with lights switched on at 6:00 am. All procedures took place during the light phase. Anesthesia was initially induced by placing the mice for 3–5 min in a chamber filled with 3% volume-to-volume isoflurane (Aesica Pharmaceuticals, Hertfordshire, UK). The mice were then positioned on a warm pad (ALA Scientific Instruments, New York, NY, USA) that maintained their temperature during ECG recording. The mice were able to breath freely through a nose cone. Anesthesia was maintained by inhalation of 1.5% isoflurane. Continuous 5-min ECGs were obtained using subcutaneous electrodes attached to the four limbs and recorded via a PowerLab data acquisition system (model ML866, ADInstruments, Colorado Springs, CO, USA) and Animal Bio Amp (model ML136, ADInstruments). The ECG analysis was performed in an unbiased fashion with 1500 beats being analyzed using LabChart 7 Pro version 7.3.1 (ADInstruments). Detection and analysis of QTc interval, QRS intervals, Tpeak-Tend intervals were set to Mouse ECG parameters. The values obtained were compared statistically by the Mann–Whitney U test, and a *p* < 0.05 was accepted as significant.

### Western blotting

Skeletal muscle (femoris and gastrocnemius) and cardiac muscle tissue samples were homogenized using a MagNA Lyser (Roche, Basel, Switzerland) in RIPA buffer (50 mM Tris at pH 7.4, 150 mM NaCl, 1 mM EDTA, 1% Triton X-100, 0.5% Sodium deoxycholate, 0.1% SDS with complete protease inhibitor and phosphatase inhibitor cocktails [Roche, #04693124001]) and then denatured in 2% SDS sample buffer (50 mM Tris at pH 6.8, 100 mM Dithiothreitol, 2% SDS and 10% glycerol) for 15 min at 100 °C. Total protein lysate was separated by SDS-polyacrylamide gel electrophoresis (Bio-Rad, Hercules, CA, USA) and then electro-transferred to a polyvinylidene fluoride transfer membrane (PerkinElmer, Waltham, MA, USA, #NEF1002001PK). The membrane was blocked with 5% (w/v) non-fat dried milk in TBST buffer (25 mM Tris at pH 7.5, 137 mM NaCl, 2.7 mM KCl and 0.1% Tween-20 [v/v]) for 1 h at room temperature, and then incubated with a primary antibody for 14–16 h at 4 °C. The membrane was then washed three times with TBST buffer before probing with an appropriate secondary antibody for 1 h at room temperature; washing and then detection by ECL (Thermo Fischer Scientific, Waltham, MA, USA, #34580). The following antibodies were used: Cisd2, Gapdh (Millipore, Burlington, MA, USA, #MAB374), Anti-Rabbit IgG HRP Linked (Sigma-Aldrich, #NA934) and Anti-Mouse IgG HRP Linked (Sigma-Aldrich, #NA931).

### Histopathology and transmission electron microscopy (TEM)

Mouse skeletal muscle (femoris and gastrocnemius) and cardiac muscle tissue samples were harvested and then fixed with 10% formalin for 14–16 h at 4 °C. The samples were processed using a tissue processor (STP120, MICROM, Walldorf, Germany) and embedded in paraffin. H&E, Masson’s trichrome and Sirius Red staining of tissue sections (3–4 μm) were carried out by standard protocols [22]. The TEM was performed as described previously [24]. In brief, mouse skeletal muscle (gastrocnemius) and cardiac muscle tissues were fixed in a TEM fix buffer (1.5% glutaraldehyde and 1.5% paraformaldehyde in 0.1 M cacodylate buffer at pH 7.3), post-fixed in 1% OsO_4_ and 1.5% potassium hexanoferrate and then tissues were washed in cacodylate and 0.2 M sodium maleate buffers (pH 6.0) followed by block-stained with 1% uranyl acetate. Following dehydration, the skeletal muscle (gastrocnemius) tissue and the cardiac muscle tissue were embedded in Epon (EMS, Hatfield, PA, USA, #14120) and sectioned for TEM analysis.

### Tissue reactive oxygen species (ROS) and reactive nitrogen species (RNS) levels

ROS and RNS levels were assayed in the skeletal muscle and cardiac muscle tissue lysates by an in vitro ROS/RNS Assay Kit for quantification of ROS and RNS levels following the manufacturer’s instructions (Cell Biolabs, San Diego, CA, USA, #STA-347). The fluorescence intensity of 2ʹ,7ʹ-dichlorodihydrofluorescein (DCF) was monitored using an Infinite 200 Microplate Reader (Tecan Group Ltd.).

### Tissue RNA isolation, RNA sequencing, and pathway analysis

Total RNA was isolated from skeletal muscle (gastrocnemius), cardiac muscle, and liver tissue using TRI Reagent (Sigma-Aldrich, #T9424) and phenol/chloroform extraction. The quality of the total RNA was examined using an Agilent 2100 Bioanalyzer (Agilent Technologies, Santa Clara, CA, USA); samples with an RNA Integrity Number higher than 8 were subjected to RNA sequencing. The RNA sequencing (RNA-seq) was conducted by the Genome Research Center at National Yang Ming Chiao Tung University. The dataset was generated to a depth of at least 20 million reads for each sample by single-end sequencing. After mapping, the unique gene reads were analyzed as RPKM (reads per kilobase of exon model per million reads) to assess gene expression. A total of 6404 and 6231 genes were retained after filtering to identify expressed genes in the cardiac muscle and skeletal muscle (gastrocnemius) tissues (minimal counts in RPKM > 4 detected in at least 50% of samples), respectively. The *p*-values of the gene expressions were adjusted using the Benjamini–Hochberg method. Differentially expressed genes (DEGs) were identified using a false discovery rate (FDR) cut-off threshold as indicated in the figure legends. DEGs reversed by hesperetin were analyzed using the following criteria: (1) 26-month WT-Veh vs. 3-month WT, FDR < 0.1; (2) 26-month WT-hesperetin vs. 26-month WT-Veh, *p* < 0.05, and reversing of 26-month WT-Veh vs. 3-month WT; (3) 26-month WT-hesperetin vs. 3-month WT, *p* > 0.05. The DEGs from the RPKM was loaded into the EZinfo software package for principal component analysis (PCA, EZinfo 3.0.3 software, Umetrics, Umeå, Sweden). Gene Ontology (GO) functional characterization was performed using the online tools PANTHER (www.pantherdb.org) and Mouse Genome Informatics (MGI) GO term finder (www.informatics.jax.org). The values of the RPKM were transformed into z-scores and these scores were used to generate heatmaps using Multi Experiment Viewer 4.9 software (mev.tm4.org).

### Statistical analysis

The data are presented as mean ± SD or mean ± SEM, as described in the figure legends. Comparisons between two groups were carried out using an unpaired two-tailed Student’s t test. Comparisons among groups greater than two were carried out using either one-way or two-way ANOVA with Bonferroni multiple comparison test as indicated in the figure legends. The survival rates of the mice were compared using a log-rank (Mantel–Cox) test; power analysis (SPSS Statistics 26.0, IBM Corp, Armonk, NY, USA) revealed that a sample size of 47 animals (including the no-treatment, Veh and hesperetin groups) has a power of 0.9608. When analyzing statistical differences among groups, *p* < 0.05 was considered significant using the software Graphpad Prism 6.0 (GraphPad Software, San Diego, CA, USA).

## Results

### Hesperetin is a promising Cisd2 activator with no detectable toxicity

The beneficial effects of Cisd2 on slowing down aging in mice have prompted us to translate the genetic evidence into a pharmaceutical application. To this end, we established a HEK293-CISD2 reporter cell line and a transgenic (TG) reporter mouse model using the human BAC (bacterial artificial chromosome) clone CTD-2303J4 that contains the intact gene of human CISD2, in order to systematically screen for CISD2 activators capable of enhancing CISD2 expression at the transcriptional level (Additional file 1: Fig. S1A, B). We screened a herb compound library that had been established by our team from 60 noble herbs (Additional file 1: Table S1); these herbs were described in a traditional Chinese medicine handbook, *The Divine Husbandman's Herbal Foundation Canon* [35]. After substantial fractionation and characterization, as well as bioassay-guided analyses of various structural analogs of the identified flavonoids, we identified hesperetin (a single compound with > 98% purity) as a promising Cisd2 activator that is able to enhance Cisd2 expression both in vitro and in vivo (Additional file 1: Fig. S1C–G).

Importantly, hesperetin has no detectable toxicity under the conditions that are effective in HEK293 cells (10–30 μM) and in WT young and old mice. We provided hesperetin in the mice’s food (100 mg/kg/day) in order to treat naturally aged mice during old age. The dose used in this study is an achievable dose in humans and is a human equivalent dose of 491 mg/60 kg/day based on an equation developed for interspecies dose conversion from animal to human studies [42]. After 6 months of treatment of the old WT mice (aged from 20- to 26-month old), serum biochemical analyses revealed that hesperetin has no detectable toxicity compared with a sex-matched and age-matched control group treated with Veh. The in vivo serum parameters analyzed included (1) damage related to liver, kidney, and cardiac functions; (2) metabolic indices for serum insulin, total cholesterol, and triglycerides; and (3) various electrolytes, namely Ca^2+^, Mg^2+^, Na^+^, K^+^, and Cl^−^ ions. Interestingly, hesperetin seems to decrease a liver injury marker, namely aspartate aminotransferase (Additional file 1: Fig. S2). Additionally, a CBC analysis revealed that hesperetin has no detectable toxicity on hematological parameters after 7 months of treatment of old WT mice (Additional file 1: Fig. S3). Taken together, these serum biochemical and CBC analyses confirm that hesperetin has no detectable toxicity when used for the long-term treatment of old mice.

Furthermore, we have quantified the compound concentrations of hesperetin and two of its major conjugated metabolites, namely H7G and H7S, in the serum, liver, skeletal muscle and heart of the dietary hesperetin-treated mice (Additional file 1: Fig. S4A–D). In the serum and tissues examined, all of the three compounds (hesperetin, H7G and H7S) are detectable. Interestingly, both hesperetin (10 μM) and a higher concentration of H7S (30 μM) are able to activate the CISD2 reporter to a similar level in the HEK293-CISD2 reporter cells (Additional file 1: Fig. S4E). Accordingly, the anti-aging effects of dietary hesperetin could be a combined activity of hesperetin and its conjugated metabolites, such as the H7S.

### Hesperetin delays aging and promotes longevity in naturally aged WT mice

**Fig. 1:**
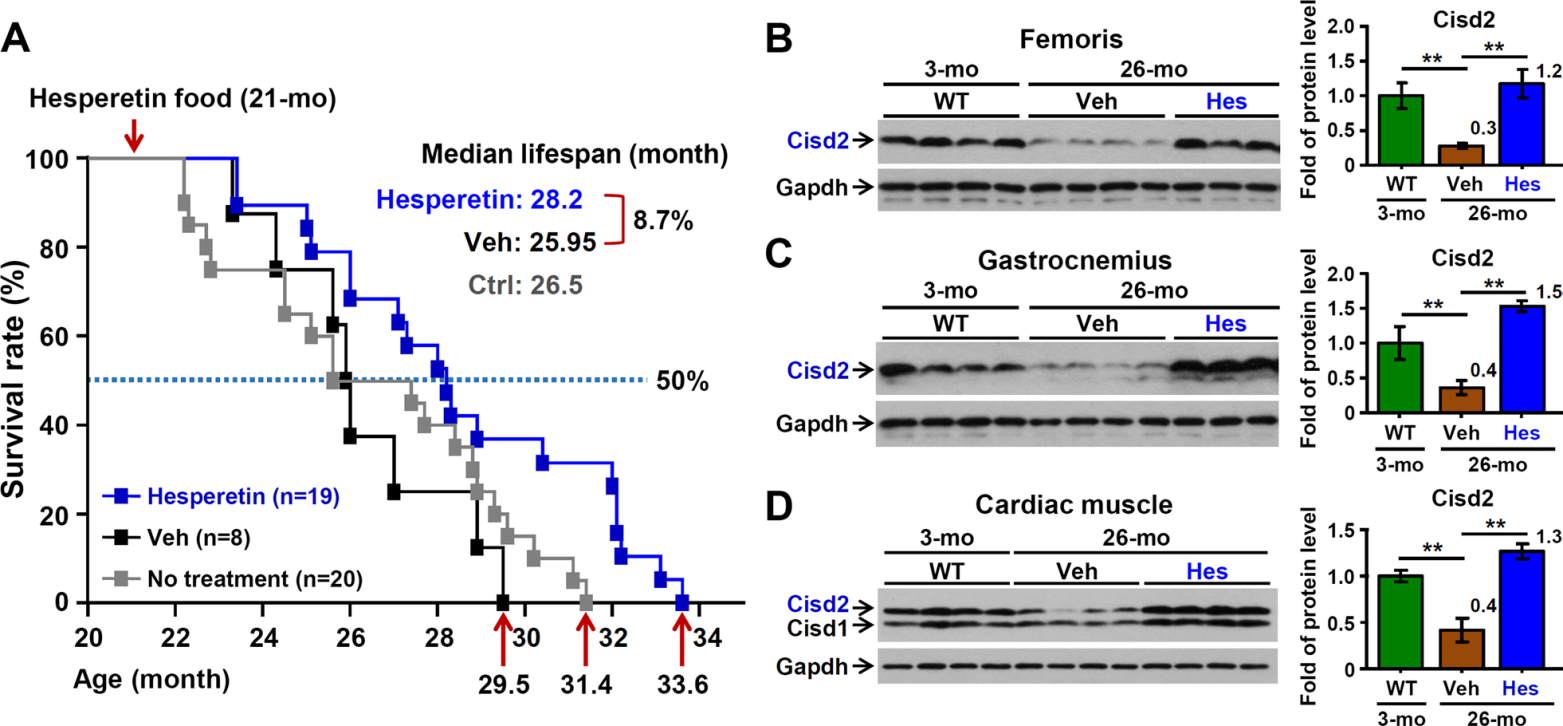
Hesperetin extends lifespan and enhances Cisd2 levels in old WT mice. **A** The survival rate of WT mice without any treatment (n = 20 mice) and old WT mice treated with vehicle (Veh; n = 8) or hesperetin (Hes; n = 19). For the hesperetin treatment, 21-month old WT mice were treated with dietary hesperetin (100 mg/kg/day) or Veh control food. Statistical comparison of the survival curves by log-rank test: Veh versus hesperetin, *p* = 0.04; no treatment WT versus hesperetin, *p* = 0.029; no treatment WT versus Veh, *p* = 0.5 (n.s.). **B**–**D** Elevation of Cisd2 protein levels in multiple tissues of old mice fed with dietary hesperetin at old age. Western blot of Cisd2 in the femoris (**B**), gastrocnemius (**C**), and cardiac muscles (**D**) of 26-month mice treated with dietary hesperetin or Veh control food for 5 months (from 21-month old). The protein level of Cisd2 in 3-month WT mice serves as a young mouse control. Data are presented as mean ± SD. *p < 0.05; **p < 0.005 by one-way ANOVA with Bonferroni multiple comparison test. All the mice used in this study are males

To investigate if hesperetin is able to slow down aging and extend a healthy lifespan, we treated naturally aged mice started at 21-month old with dietary hesperetin and monitored their survival rate. Intriguingly, hesperetin significantly extends the lifespan of the aging mice (Fig. 1A). The median lifespan of the hesperetin-treated WT mice was increased by 2.25 months (8.7%; from 25.95- to 28.2-month old) relative to Veh-treated WT mice (*p* = 0.04), with the maximum lifespan increase being 4.1 months (13.9%; from 29.5- to 33.6-month old). The level of Cisd2 was found to be significantly decreased in the skeletal (femoris and gastrocnemius) and cardiac muscles at 26-month in the untreated old mice. Remarkably, dietary hesperetin increases Cisd2 levels in aged tissues to a level comparable to that of young mice at 3-month old (Fig. 1B–D). These results show that Cisd2 is able to be targeted pharmaceutically and that it is activated by dietary hesperetin during the late stage of life. As a result of the enhanced level of Cisd2, there appears to be a delay in aging and a prolongation of the lifespan among naturally aged mice.

### Hesperetin attenuates whole-body metabolic decline in old mice

**Fig. 2:**
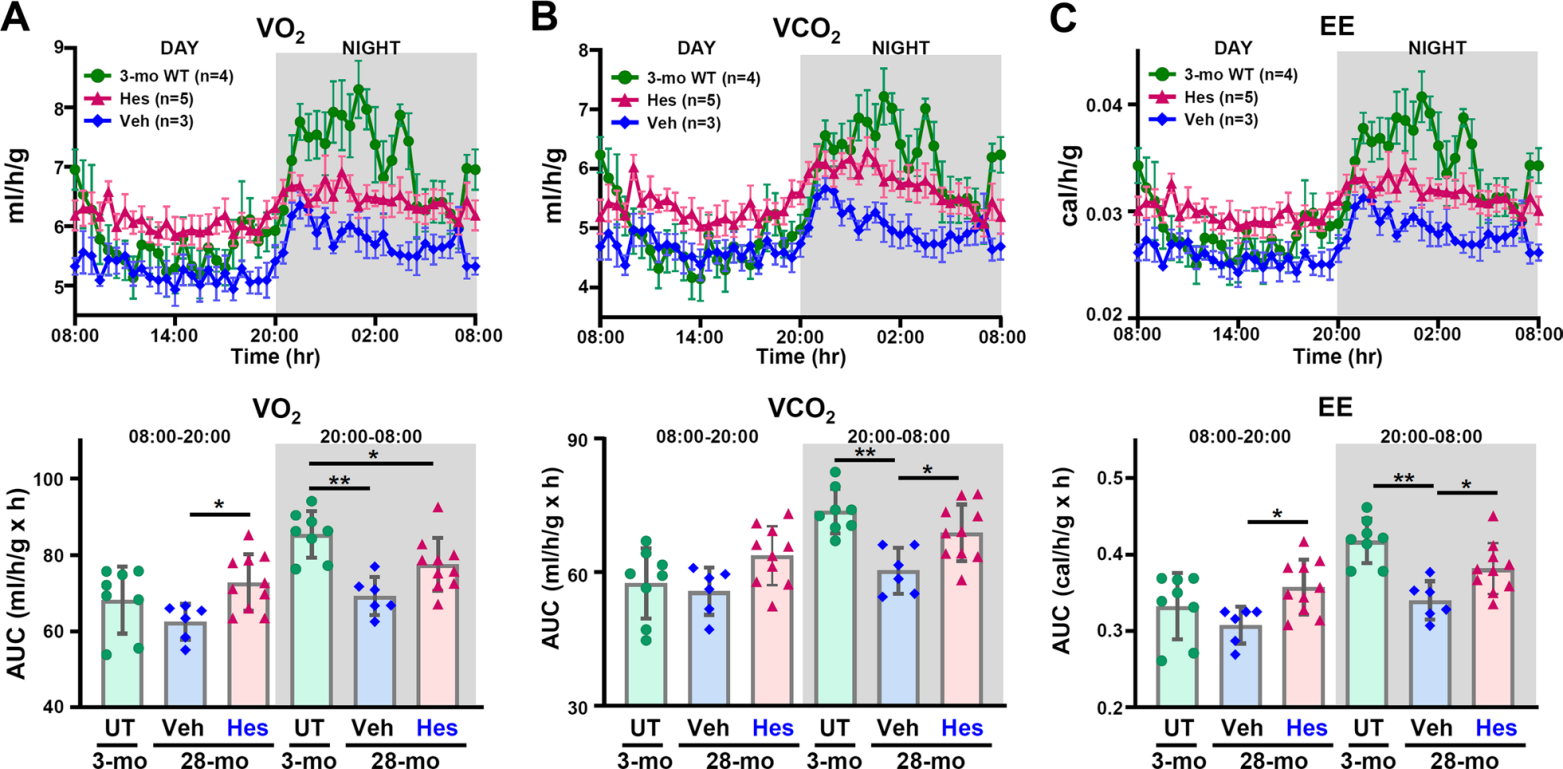
Hesperetin attenuates whole-body metabolic decline in old WT mice. Hour-to-hour average and quantification of **A** whole-body oxygen consumption (VO_2_), **B** CO_2_ production (VCO_2_), and **C** energy expenditure (EE) during the light and dark periods for 3-month WT, 28-month vehicle (Veh) treated and 28-month hesperetin (Hes) treated WT mice. The old mice (22-month old) were treated with dietary hesperetin (100 mg/kg/day) or Veh control food for 6 months and monitored at 28-month old. The metabolic rate of an individual mouse was monitored for 48 h. The area under the curve (AUC) from 20:00 to 02:00 during dark period is quantified. For each mouse, two AUC quantitative values of each metabolic index calculated from the data of two cycles of 24-h measurement are presented. Data for the VO_2_, VCO_2_, and energy expenditure are normalized to lean mass. Data are presented as mean ± SEM in the hour-to-hour metabolic monitoring. Data for quantification of AUC are presented as mean ± SD. *p < 0.05; **p < 0.005 by one-way ANOVA with Bonferroni correction for multiple comparisons. *UT* untreated

The hallmarks of aging are tightly associated with metabolic alterations [43]. Decreased whole-body metabolism, increased body fat accumulation, loss of muscle mass and impaired glucose homeostasis are all observed during aging [44]. To study the anti-aging and beneficial effects of hesperetin on age-related metabolic decline, we examined the whole-body energy metabolism and body composition of the old mice treated with hesperetin. Our results reveal that the VO_2_, CO_2_ and EE of Veh-treated old (28-month old) WT mice were significantly decreased compared to young (3-month old) WT mice. Importantly and intriguingly, hesperetin treatment for 6 months attenuates these age-related reductions that affect the whole-body metabolism of the old mice during their dark period (Fig. 2A–C). When compare over the duration of the treatment, among the hesperetin-treated group, the older 28-month mice (hesperetin treatment for 6 months) seem to show a trend towards a higher metabolic rate than that among the 23.5-month mice (hesperetin treatment for 1.5 months) (Additional file 1: Fig. S5). Taken together, these metabolic analyses reveal that hesperetin appears to attenuate the age-related metabolic decline of old mice.

### Hesperetin reduces fat and improves glucose homeostasis in old mice

**Fig. 3:**
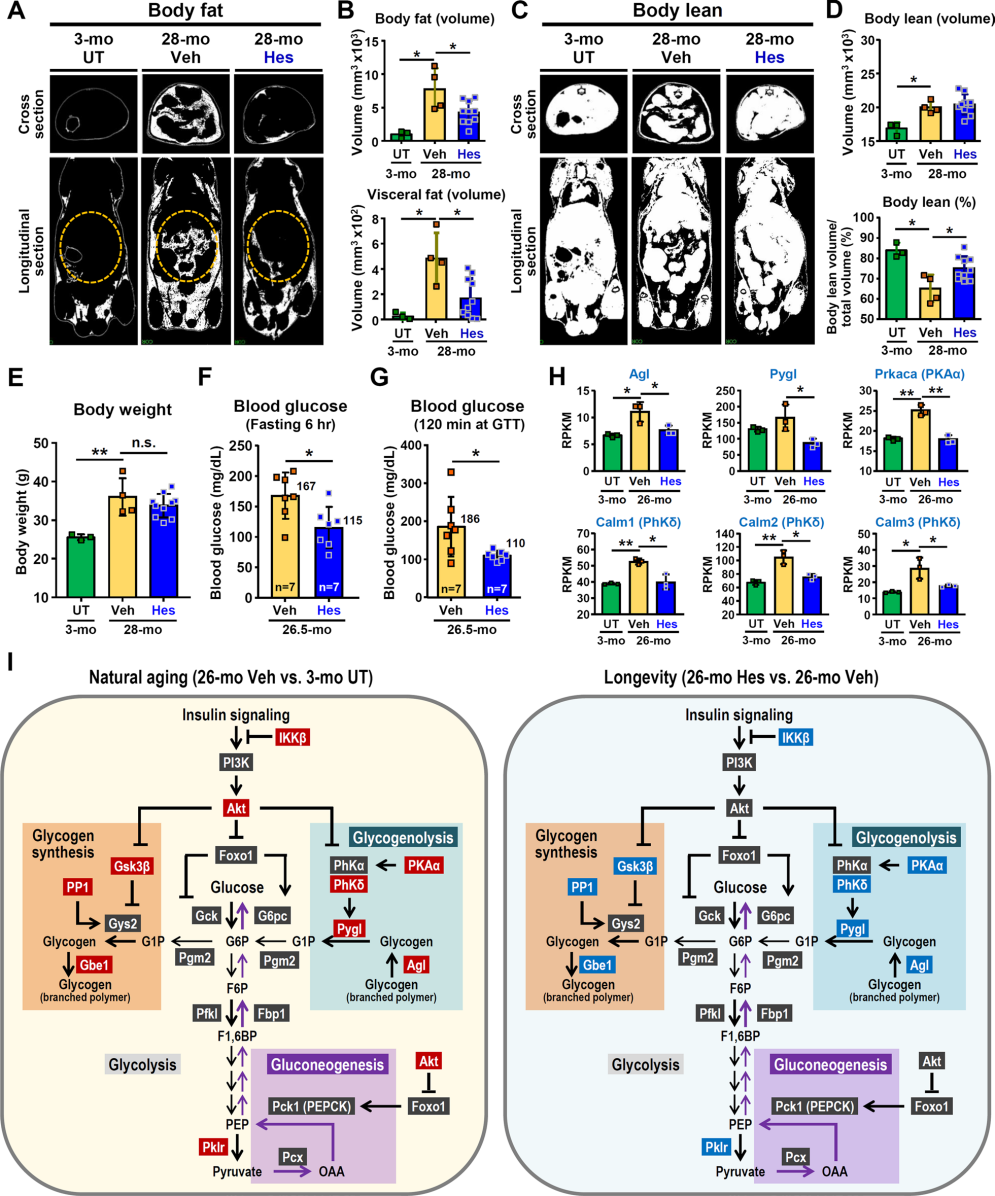
Hesperetin treatment reduces fat and improves glucose homeostasis in old WT mice. **A**–**D** Hesperetin treatment for 6 months (from 22- to 28-month old). **A**, **B** Representative Micro-CT analysis and quantification of total body fat (volume) and visceral fat (volume) in 3-month WT, 28-month Veh-treated and 28-month hesperetin-treated WT mice (n = 3–10 mice per group). Yellow circles indicate the area of visceral fat for quantification. **C**, **D** Representative Micro-CT analysis and quantification of total body lean (volume) and percent of body lean (%) in 3-month WT, 28-month Veh-treated and 28-month hesperetin-treated WT mice (n = 3–10 mice per groups). **E** Body weight in 3-month WT, 28-month Veh-treated and 28-month hesperetin-treated WT mice (n = 3–10 mice per groups). **F**, **G** Basal levels of blood glucose (fasting 6 h), and blood glucose levels at 120 min measured during glucose tolerance test (GTT) after hesperetin treatment for 6 months (from 20.5- to 26.5-month old) in the Veh-treated and hesperetin-treated mice. **H** The mRNA levels of key enzymes involved in the pathway of glycogenolysis in the livers of 3-month WT, 26-month Veh-treated and 26-month hesperetin-treated WT mice (n = 3 mice per group) after hesperetin treatment for 5 months (from 21- to 26-month old). The mRNA levels were quantified by RNA-seq analysis. **I** Schematic pathway of the enzymes and metabolites involved in hepatic insulin signaling and glucose metabolism, including glycolysis, glycogen synthesis, gluconeogenesis, and glycogenolysis, in naturally aged Veh-treated and hesperetin-treated WT mice. The nuclear factor kappa-B kinase subunit β (IKKβ) is able to inhibit insulin signaling via phosphorylation of insulin receptor substrate 1. AKT is central to regulating hepatic insulin action and glucose metabolism. Glycolysis: The key enzymes involved in glycolysis are glucokinase (Gck), phosphofructokinase (Pfkl) and pyruvate kinase (Pklr). Glycogen synthesis: The key enzymes involved in glycogen synthesis are glycogen synthase (Gys2) and glycogen branching enzyme (Gbe1). In addition, glycogen synthase kinase 3β (Gsk3β) is able to phosphorylate and inhibit glycogen synthase activity, whereas the protein phosphatase 1 (PP1) is able to dephosphorylate and promote glycogen synthase activity. Gluconeogenesis: The key enzymes involved in gluconeogenesis are pyruvate carboxylase (Pcx), phosphoenolpyruvate carboxykinase (Pck1/Pepck), fructose 1,6-bisphosphatase (Fbp1) and glucose-6-phosphatase (G6pc). Glycogenolysis: The key enzymes involved in glycogenolysis are glycogen debranching enzyme (Agl) and glycogen phosphorylase (Pygl). In addition, protein kinase A alpha (PKAα) is able to phosphorylate and activate the α subunit of phosphorylase kinase (PhKα); subsequently, the activated PhK phosphorylates Pygl to increase its enzymatic activity. Phosphorylase kinase is one of the three main families of Ca^2+^/Calmodulin-dependent protein kinases. Moreover, the δ subunit of phosphorylase kinase (PhKδ) is the endogenous calmodulin, the activity of which is able to be regulated by intracellular Ca^2+^ levels. G6P, glucose-6-phosphate; F6P, fructose 6-phosphate; F1,6BP, fructose 1,6-bisphosphate; PEP, phosphoenolpyruvate; OAA, oxaloacetate; G1P, glucose 1-phosphate; Pgm2, phosphoglucomutase 2. Results are presented as mean ± SD. *p < 0.05; **p < 0.005; not significant (n.s.). In (**B**), (**D**), (**E**) and (**H**), the statistical analyses were performed by one-way ANOVA with Bonferroni multiple comparison test. In (**F**) and (**G**), the statistical analyses were performed by Student’s t test. All the mice used in this study are males. *UT* untreated

Body composition analysis, in particular the fat and muscle present in the whole body, can be used to evaluate physical fitness. Interestingly, when body composition was investigated, our results reveal that total body fat and visceral fat are significantly increased in Veh-treated old (28-month) WT mice compared with young (3-month) WT mice. Notably, dietary hesperetin treatment for 6 months attenuates this age-related body fat and visceral fat accumulation (Fig. 3A, B). Moreover, hesperetin treatment also attenuated age-related muscle loss; specifically, the percentage of bodily lean mass is significantly higher in the hesperetin-treated old mice compared with that in the Veh-treated control group (Fig. 3C, D). However, no significant difference in body weight was found (Fig. 3E).

Furthermore, impaired glucose homeostasis and insulin resistant are also hallmarks of age-related metabolic alterations and are highly associated with the age-related decreases in skeletal muscle mass [44]. To study the beneficial effect of hesperetin on glucose homeostasis in old mice, we perform glucose tolerance tests (GTTs) and insulin tolerance tests (ITTs). There is no significant difference in the GTTs and ITTs between the Veh-treated and hesperetin-treated old mice (Additional file 1: Fig. S6); however, there seems to be a trend towards better GTT performance among the hesperetin-treated old mice. In addition, a previous study has revealed that, in humans, there is a positive correlation between aging and progressive elevation of blood glucose, including fasting and 120-min of GTT blood glucose levels [45]. In our mouse study, notably, after hesperetin treatment for 6 months, there was in a significant decrease in both fasting (6 h) and 120-min of GTT blood glucose levels (Fig. 3F, G).

To study the potential mechanism involved, we performed hepatic transcriptome RNA sequencing analysis in order to examine the expression levels of the key genes involved in the insulin signaling pathway and in the regulation of glucose homeostasis, namely the pathways of glycolysis, gluconeogenesis, glycogen synthesis, and glycogenolysis. Insulin promotes glycogen synthesis and inhibits both glycogenolysis and gluconeogenesis via the regulation of multiple pathways downstream of insulin; this includes activation in the liver of PI3K/Akt signaling, inhibition of glycogen synthase kinase 3β (GSK3β) and inhibition of forkhead box protein O1 (Foxo1) [46, 47]. Previous studies have revealed that dysregulation of hepatic insulin signaling results in impaired glucose homeostasis and the development of insulin-resistance during aging [48]. Notably, in the Veh-treated old mice, most of the DEGs related to insulin signaling pathway are significantly increased in the livers; most of these DEGs belong to four major functional groups, namely inhibition of insulin signaling, glucose metabolism, lipid metabolism, and cell proliferation/differentiation (Additional file 1: Fig. S7A). Interestingly, in the hesperetin-treated old mice, the expression of the DEGs related to insulin signaling pathway and glucose metabolism show a reversal towards an expression level similar to that of young mice (Additional file 1: Fig. S7B–D). Importantly, the expression of nuclear factor kappa-B kinase subunit β (IKKβ), which inhibits insulin signaling and is associated with hepatic insulin resistance [49], is significantly increased in the livers of the Veh-treated old mice and this is reverted by hesperetin treatment (Additional file 1: Fig. S7B), which suggests that hesperetin appears to improve hepatic insulin sensitivity.

Intriguingly, four key enzymes involved in the hepatic glycogenolysis, namely glycogen debranching enzyme (Agl), glycogen phosphorylase (Pygl), protein kinase A alpha (PKAα), and δ subunit of phosphorylase kinase (PhKδ), appear to be playing important roles in modulating the levels of blood glucose after hesperetin treatment (Fig. 3I). Agl and Pygl are key enzymes and are involved in hepatic glycogenolysis to produce glucose. PKAα is a positive regulator that enhances glycogenolysis and it is able to phosphorylate and activate the α subunit of phosphorylase kinase (PhKα). PhKδ, calmodulin, is encoded by five different genes (Calm1, Calm2, Calm3, Calm4 and Calm5) in mice; however, the expression of Calm4 and Calm5 was not detectable in the liver by RNA sequencing. PhKδ regulates PhK enzyme activity in a Ca^2+^-dependent manner [50, 51]. Subsequently, the activated PhK phosphorylates Pygl to switch on its enzymatic activity thus increasing the production of glucose (Fig. 3I). In the Veh-treated old mice, these four key enzymes of glycogenolysis (Agl, Pygl, PKAα, and PhKδ) are significantly increased. Remarkably, in the hesperetin-treated old mice, the elevation of these enzymes is down-regulated and reversed to give a level of expression comparable to that of the young mice (Fig. 3H, I); as a consequent it seems that the elevation in blood glucose found in old untreated mice is attenuated. A similar observation indicating that age-related impairment of hepatic glucose production can be prevented by the suppression of glycogenolysis, thereby down-regulating the blood glucose, has been reported previously [52].

When glycogen synthesis is explored, there is known to be two key enzymes that are involved in this process, namely glycogen synthase (Gys2) and glycogen branching enzyme (Gbe1) (Fig. 3I). Insulin signaling enhances Gys2 activity via activation of protein phosphatase 1 (PP1), which is encoded by Ppp1ca gene, and by inhibition of glycogen synthase kinase 3β (Gsk3β) [46]. PP1, which is a positive regulator that enhances glycogen synthesis. The latter enzyme can dephosphorylate and this activates Gys2. However, Gsk3β is a negative regulator and is able to inhibit glycogen synthesis because it can phosphorylate and inhibit Gys2. In the Veh-treated old mice, these key enzymes of glycogen synthesis (Gsk3β, PP1, and Gbe1) are significantly increased. Remarkably, in the hesperetin-treated old mice, their expression is significantly down-regulated and have reverted to an expression level comparable to that in the young mice (Fig. 3I; Additional file 1: Fig. S7E, F). However, there is no significant difference in the gluconeogenesis pathway between the livers of Veh-treated and hesperetin-treated old mice. In summary, our hepatic transcriptomic analyses reveal that hesperetin appears to ameliorate age-related dysregulation of insulin signaling in the liver and this improve glucose homeostasis in the treated old mice via the maintenance of normal glycogen metabolism including both glycogenolysis and glycogen synthesis.

### Hesperetin slows down skeletal muscle aging in old mice

**Fig. 4:**
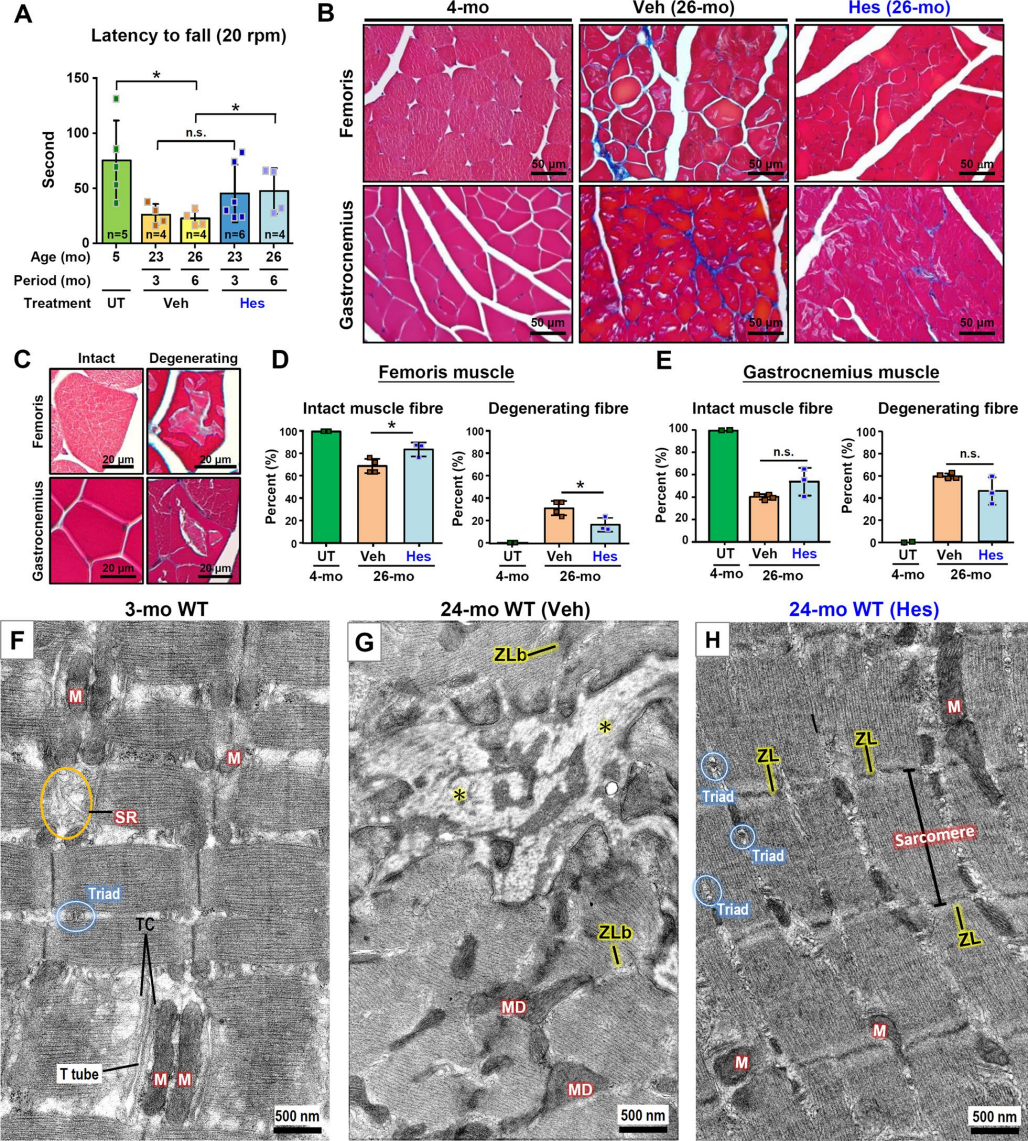
Hesperetin slows down skeletal muscle aging in old WT mice. **A** The Rotarod tests in old WT mice (n = 4–7 mice) were carried out after dietary hesperetin (100 mg/kg/day) or vehicle control food treatment for 3 and 6 months (started at 20-month old). **B** Masson’s trichrome staining of femoris and gastrocnemius muscles. The 21-month old WT mice were treated with dietary hesperetin for 5 months and sacrificed at 26-month old. **C** The representative micrographs showing muscle fibers with an intact and a degenerated morphology in the femoris and gastrocnemius muscles. **D**, **E** Quantification of intact and degenerating muscle fibers in femoris and gastrocnemius. Data are presented as mean ± SD. *p < 0.05; **p < 0.005 by one-way ANOVA with Bonferroni multiple comparison test in (**D**) and (**E**) or Student’s t test in (**A**). **F**–**H** TEM analysis of gastrocnemius muscle. **F** Young mice at 3-month old. **G** Veh-treated mice at 24-month old. Mitochondrial degeneration (MD) and fibrosis (*), which may be caused by myofibril degeneration and Z-line breakdown (ZLb), are evident and can be easily detected in the Veh-treated WT mice at 24-month old. **H** Hesperetin-treated mice at 24-month old. The age-related degeneration appears to be reversed as revealed by the presence of intact Z-lines (ZLs) and multiple normal-sized triads. M, mitochondria; SR, sarcoplasmic reticulum; TC, terminal cisternae of the SR. Scale bars, 500 nm

Sarcopenia, which is characterized by the degenerative loss of skeletal muscle mass and strength, is accompanied by mitochondrial degeneration and is a hallmark of skeletal muscle aging; these changes compromise the functions of the skeletal muscles [44, 53]. To study the anti-aging effects of hesperetin on skeletal muscles, we evaluate the function and structure of skeletal muscles in the old mice treated with hesperetin. Notably, hesperetin treatment alleviates the age-related functional decline of skeletal muscles, as revealed by rotarod tests. Interestingly, the functional analysis by rotarod test revealed that, in the hesperetin-treated group, the 26-month WT mice (hesperetin treatment for 6 months) were found to have a better performance compared with the control WT mice at 26-month old (Veh treatment for 6 months) (Fig. 4A). In addition, fibrosis analysis using Masson’s trichrome staining revealed overt degeneration and fibrosis of the skeletal muscles of the old control WT mice (26-month old); however, hesperetin was able to alleviate these age-related histopathological deleterious changes of the skeletal muscles (Fig. 4B). Furthermore, a significant decrease in the percentage of degenerating muscle fibers were found to be present in both the femoris and gastrocnemius muscles was observed for the hesperetin-treated old mice compared to the Veh treated control old mice (Fig. 4C–E).

When we used TEM to analyze the ultrastructure of skeletal muscles we found disturbance of triad architecture, which involves a T tubule surrounded by terminal cisterna on either side, as well as degeneration of mitochondria, in the Veh-treated old mice compared to young mice at 3-month old. Furthermore, myofibril degeneration, Z-line breakdown and overt fibrosis, were also detectable in the Veh-treated old mice (Fig. 4F, G). Strikingly, hesperetin treatment for 3 months appears to attenuate and partially reverse these age-related deleterious effects at the ultrastructural level, namely there is preservation of the triad architecture and sarcomere, as well as a decrease in mitochondrial degeneration. This means there is a move toward a younger skeletal muscle ultrastructural pattern among the hesperetin-treated old mice (Fig. 4H).

### Hesperetin slows down cardiac aging in old mice

**Fig. 5:**
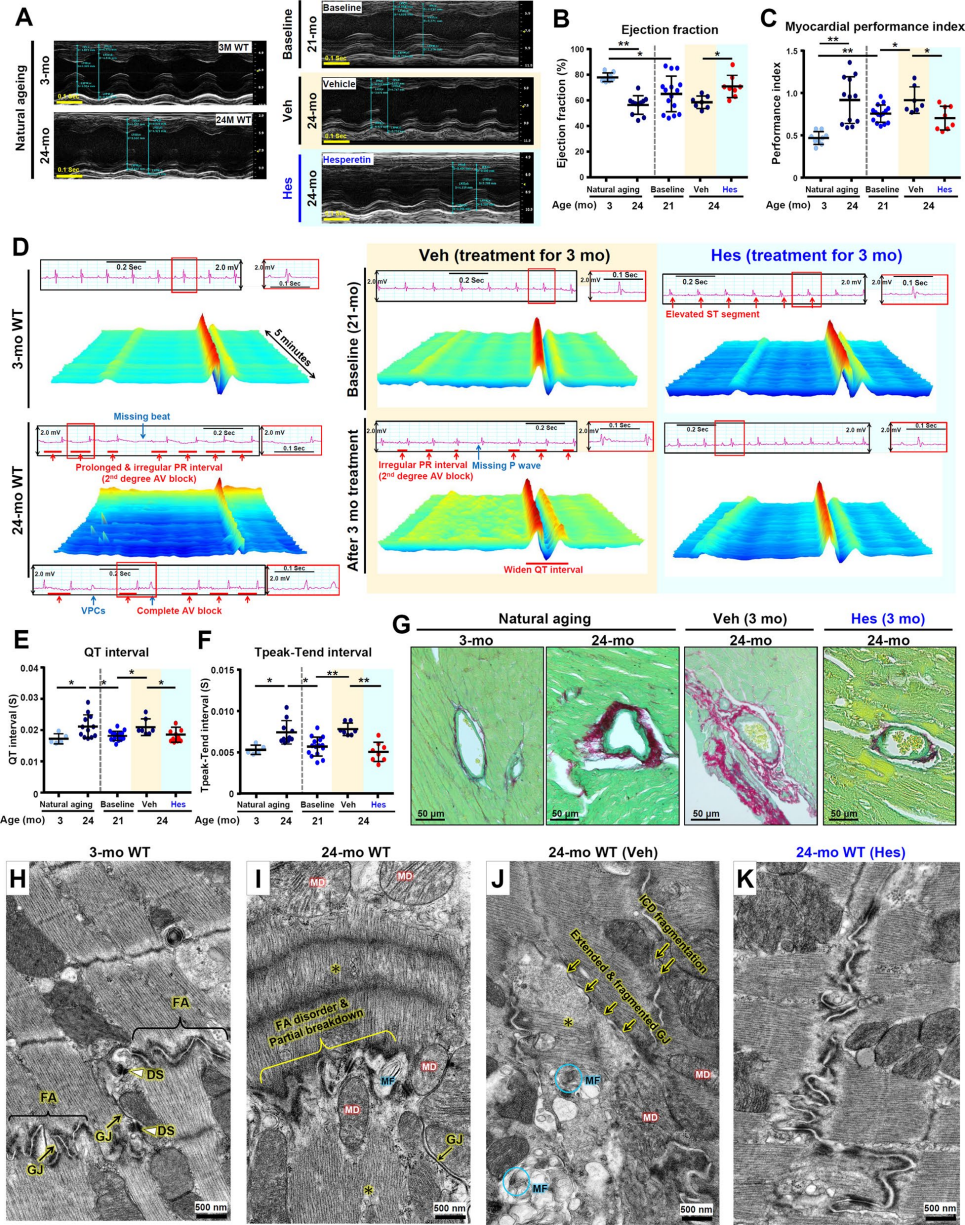
Hesperetin slows down cardiac aging in old WT mice. **A** Representative echocardiography images are shown in different groups of mice (n ≥ 5 per group). **B**, **C** Ejection fraction and myocardial performance index obtained from echocardiography analysis. **D** Representative ECG tracings and continuous 5-min waterfall plots recorded following anesthesia of the mice. Representative dysrhythmic ECGs, namely missing beat, ventricular premature complexes (VPCs), atrioventricular block (AV block), irregular PR interval, and widened QT interval, were found in the old WT mice or Veh-treated mice. **E**, **F** QT interval and Tpeak-Tend interval measurements obtained from 5-min sequential beats of whole ECG tracings from baseline. **G** Cardiac perivascular fibrosis is examined by Sirius Red/Fast Green staining of the hearts with the aim of detecting collagen. **H**–**K** TEM analysis reveals ultrastructure of the mitochondria, myofibril, and ICD in the cardiac muscle of young mice at 3-month (**H**), old WT mice at 24-month (**I**), Veh-treated WT mice at 24-month (**J**), and hesperetin-treated WT mice at 24-month (**K**). Overt ultrastructural abnormalities are present in the cardiac muscles of naturally aged mice or Veh-treated WT mice at 24-month old. Myelin figure (MF); FA, fascia adherens; GJ, gap junction; DS, desmosome; MF, myelin figure; MD, mitochondrial degeneration; myofibril degeneration and disorganization (*). For hesperetin treatment in this study, old WT mice at 21-month old are treated with dietary hesperetin (100 mg/kg/day) or Veh control food for 3 months and sacrificed at 24-month old. Quantified data are presented as mean ± SD and analyzed by one-way ANOVA with Bonferroni multiple comparison test. *p < 0.05; **p < 0.005

The incidence and prevalence of heart failure increases in an aged population and is without effective treatment [54]. In human elderly individuals, cardiac aging is characterized by the following abnormalities: a decreased ejection fraction, an increased cardiac performance index, an increase in arrhythmogenesis, and increased perivascular fibrosis [55–57]. Notably, in aged mice, all these phenotypes were also present in the hearts of the Veh-treated WT mice at 24-month old as revealed by echocardiography (measuring mechanical function), electrocardiography (ECG, measuring electrical function), and histopathological analyses (Fig. 5). Specifically, our results revealed that hesperetin improves the mechanical function of the aged heart, namely the systolic ejection fraction and diastolic myocardial performance index (Fig. 5A–C). To study the effects of hesperetin on age-related arrhythmogenesis and electrical dysregulation, we evaluate a continuous 5-min ECG trace obtained using old mice under hesperetin treatment. Strikingly, hesperetin appears to alleviate age-related arrhythmogenesis (Fig. 5D) and rescue abnormalities affecting the QT interval (Fig. 5E) and Tpeak-Tend interval (Fig. 5F) present in the naturally aged mice. Furthermore, cardiac perivascular fibrosis was also attenuated in the hesperetin treatment aged mice as revealed by Sirius Red/Fast Green staining of their hearts to detect the presence of collagen (Fig. 5G).

In addition, at the ultrastructural level, hesperetin ameliorates age-related deterioration of the intercalated disc (ICD), the mitochondria, and the sarcomeres present in cardiac muscle. The ICD is a structure that connects and synchronizes individual cardiomyocytes and allows them to work together. We use TEM to examine the integrity of the three types of cell junctions that make up the ICD, namely gap junctions, desmosomes, and fascia adherens. In 3-month WT mice (Fig. 5H), the three types of cell junctions were easily identified. On the other hand, in 24-month WT mice without (Fig. 5I) or with Veh treatment (Fig. 5J), severe ultrastructural alterations were detected. These include breakdown of the fascia adherens, extension and fragmentation of the gap junctions, and partial degeneration of the desmosomes with an increase in the space between the two membranes of the ICD. Additionally, degenerated and swollen mitochondria, as well as disorganized and degenerated myofibrils, were easily detected in 24-month WT mice. Interestingly, after 3 months of hesperetin treatment (Fig. 5K), the ultrastructural damage to the aged heart had largely disappeared and seem to have been reversed resulting in the ICD features and mitochondria now resembling the situation observed in the hearts of 3-month WT mice. Altogether, these results demonstrate that hesperetin, when given at a late-life stage to naturally aged mice, is able to improve cardiac electromechanical function and bring about a delay in cardiac aging.

### The anti-aging effect of hesperetin is mainly dependent on Cisd2

Importantly, we have found that the beneficial effects of hesperetin on aging and age-related phenotypes are largely dependent on Cisd2. We used Cisd2 mcKO (MCK-Cre; Cisd2f/f) mice, which carry tissue-specific Cisd2 knockouts affecting their skeletal and cardiac muscles [22], to study if hesperetin needs the presence of Cisd2 to bring about its anti-aging effects. The Cisd2 mcKO mice display an overt premature aging phenotype at 3-month old with a shortened lifespan similar to those observed in the conventional Cisd2KO mice. In addition, our previous study revealed that the aging phenotype of Cisd2 mcKO mice at 3-month old is comparable to that in the WT mice at 26-month old [22]. Accordingly, we treated Cisd2 mcKO and WT mice with hesperetin for 4 months starting at 3-month old and sacrificed them at 7-month old (Additional file 1: Fig. S8A). No significant difference in the concentration of hesperetin in their serum and various tissues, namely liver, skeletal muscle, and cardiac muscle, were found between the WT and Cisd2 mcKO mice (Additional file 1: Fig. S8B). In the skeletal muscles of the WT mice, rotarod tests and histopathological analysis revealed a normal function and well-organized structure in their muscles on treatment with Veh or hesperetin; there was no overt effect of hesperetin on these control mice since WT mice at 7-month old have a normal skeletal muscle phenotype (Additional file 1: Fig. S8C, D). In the skeletal muscles of the Cisd2 mcKO mice, in the absence of Cisd2, hesperetin did not have a beneficial effect and did not improve muscle functions and nor did it reverse the histopathological damage (Additional file 1: Fig. S8C, D). In fact, Cisd2 mcKO mice displayed overt degenerative loss and occasional rounded and shrunken fibers in their skeletal muscles both with and without hesperetin treatment (Additional file 1: Fig. S8D). Consistently, in the hearts of the Cisd2 mcKO mice, in the absence of Cisd2, hesperetin lost its beneficial effect in terms of improvements related to electrical dysfunction, namely dysrhythmic ECGs (Additional file 1: Fig. S9A, B), as well as improvements related to histopathological damage, namely myocardial injury and fibrosis (Additional file 1: Fig. S9C).

**Fig. 6:**
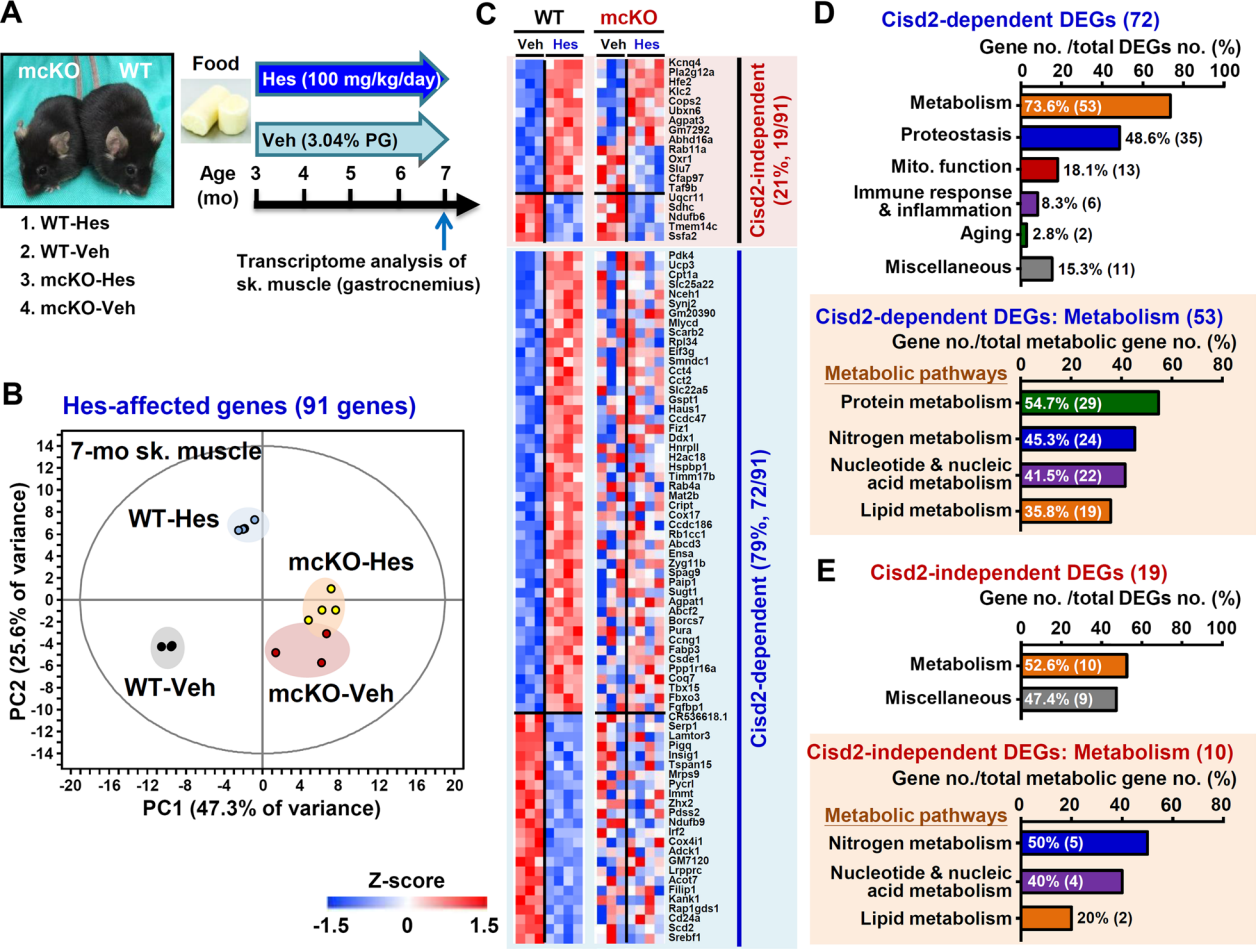
Cisd2-dependent and Cisd2-independent DEGs in the skeletal muscles of WT and Cisd2 mcKO mice after hesperetin treatment for 4 months. **A** The 3-month old WT and Cisd2 mcKO mice, which carry a Cisd2KO background specifically in the skeletal and cardiac muscles, were treated with dietary hesperetin (Hes) (100 mg/kg/day provided in food) or Veh control food (3.04% propylene glycol, w/w) for 4 months and sacrificed at 7-month old. The transcriptome of skeletal. muscle (gastrocnemius) were analyzed for the following four groups of mice: WT-hesperetin, WT-Veh, Cisd2 mcKO-hesperetin and Cisd2 mcKO-Veh. **B** Principal component analysis (PCA, EZinfo 3.0.3 software) of all the genes affected by hesperetin in the skeletal muscle of WT mice (91 DEGs, WT-hesperetin vs WT-Veh, FDR < 0.2). **C** Heatmap illustrating that the 91 DEGs (62 up-regulated and 29 down-regulated genes) can be divided into two groups: Cisd2-dependent (72/91; 79%) and Cisd2-independent (19/91; 21%). Cisd2-dependent DEGs: mcKO-hesperetin vs mcKO-Veh; p > 0.05. Cisd2-independent DEGs: mcKO-hesperetin vs mcKO-Veh; p < 0.05. **D** Pathway analysis of the Cisd2-dependent DEGs. Metabolism, which is the number one pathway, is sub-divided into four pathways, namely protein metabolism, nitrogen metabolism, nucleotide and nucleic acid metabolism, and lipid metabolism. **E** Pathway analysis of the Cisd2-independent DEGs. Metabolism, which is again the main pathway affected by hesperetin, is sub-divided into three pathways, namely nitrogen metabolism, nucleotide and nucleic acid metabolism, and lipid metabolism. The grouping of the pathways was carried out by MGI GO term finder (pathway p < 0.05)

Moreover, to differentiate the Cisd2-dependent versus Cisd2-independent effects of hesperetin, we perform transcriptomic analysis by RNA sequencing of the skeletal muscles from the following four groups of mice: WT-Hes, WT-Veh, Cisd2 mcKO-Hes and Cisd2 mcKO-Veh (Fig. 6A). DEGs analysis revealed that there are 91 DEGs (62 up-regulated and 29 down-regulated genes) affected by hesperetin in the WT mice. These DEGs can be divided into two groups: Cisd2-dependent genes (72/91; 79%) and Cisd2-independent genes (19/91; 21%) (Fig. 6B, C). Accordingly, the majority (79%) of DEGs influenced by hesperetin are Cisd2-dependent and these genes lose their differential expression patterns in the absence of Cisd2. Interestingly, there is indeed a smaller portion (21%) of the DEGs that are Cisd2-independent; these genes retain a differential expression pattern in the Cisd2 mcKO mice (Fig. 6C). Pathway analysis of these DEGs revealed that metabolism is the main pathway grouping affected by hesperetin for both the Cisd2-dependent and Cisd2-independent DEGs (Fig. 6D, E). Since Cisd2 is essential to maintaining intracellular Ca^2+^ homeostasis and mitochondrial integrity in mammals, accordingly it is not surprising that majority (72/91; 79%) of DEGs influenced by hesperetin are Cisd2-dependent and that a significant portion of them are associated with metabolism (53/72; 73.6%) and mitochondrial function (13/72; 18.1%). In summary, these results provide genetic and transcriptomic evidence confirming that hesperetin mainly exerts its anti-aging effects via the activation of Cisd2, and that it functions primarily in a Cisd2-dependent manner.

### Hesperetin treatment results in a younger transcriptome pattern

In order to gain insights into the biological effects of hesperetin on aging, we perform RNA sequencing of heart muscle and of skeletal muscle. Three sets of pair-wise DEG analyses were performed: (Set-1) 26-month WT-Veh vs. 3-month WT; (Set-2) 26-month WT-Veh vs. 26-month WT-Hes; (Set-3) 26-month WT-Hes vs. 3-month WT. A cut-off FDR of less than 0.1 was set as the significance threshold value. From these three sets of DEGs, we select genes that were differentially expressed in Set-1, that is, they showed a significant change in expression due to spontaneous aging. Using Set-2, we further selected genes that showed a significant change in expression but were reverted by hesperetin treatment. Finally, using Set-3, we further selected to identify those that were not differentially expressed between 3-month WT mice and hesperetin-treated 26-month WT mice.

**Fig. 7:**
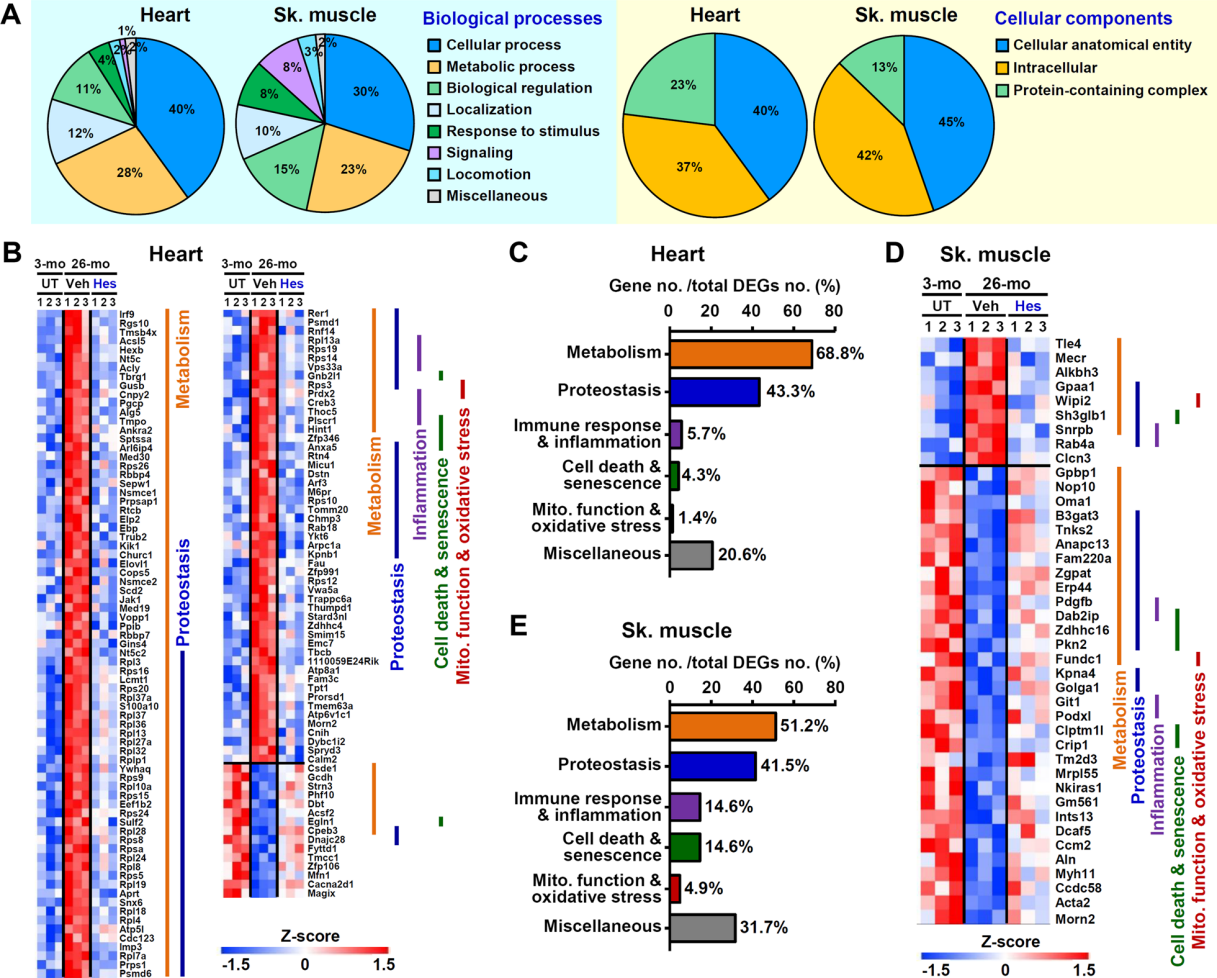
Hesperetin treatment results in a younger transcriptome pattern. **A** A pie chart shows their biological processes and subcellular localization based on GO annotation. The GO was analyzed using the PANTHER functional classification. **B** Heatmap illustrating that a total of 141 DEGs are reversed by dietary hesperetin (126 up-regulated and 15 down-regulated genes; 26-month WT-Veh vs 3-month WT, FDR < 0.1) and the aged heart pattern is moved toward the pattern of a young heart. **C** The DEGs in the aged heart, which are reverted by hesperetin in panel (**B**), are grouped into different age-related pathways and presented as a percentage. **D** Heatmap illustrating that all 41 DEGs that are reversed by dietary hesperetin (9 up-regulated and 32 down-regulated genes; 26-month WT-Veh vs 3-month WT, FDR < 0.1) and the aged skeletal muscle (gastrocnemius); this showing a movement toward the pattern present in young skeletal muscle. **E** The DEGs in the aged skeletal muscles, which are reverted by hesperetin in panel (**D**), are grouped into different age-related pathways and presented by percentage. In this study, mice were treated with dietary hesperetin (100 mg/kg/day) or Veh control food from 21- to 26-month. The grouping of the pathways was carried out by MGI GO term finder (pathway *p*-value < 0.05)

GO classification reveals that most of the DEGs reverted by hesperetin in aged heart and skeletal muscle are involved in similar biological processes and cellular components (Fig. 7A). This indicates the presence of common altered functional pathways in the cardiac and skeletal muscles of naturally aged mice. The DEGs were then further grouped into different age-related pathways by the MGI GO term finder. In both the heart (Fig. 7B, C) and skeletal muscle (Fig. 7D, E), the top five altered pathways are involved in metabolism, proteostasis, mitochondrial function and oxidative stress, cell death and senescence, as well as the immune response and inflammation. Furthermore, the subgroups of the top two pathways, namely metabolism (Additional file 1: Fig. S10) and proteostasis (Additional file 1: Fig. S11), are also similar. Additionally, hesperetin decreases the levels of reactive oxygen species (ROS) and reactive nitrogen species and shifts the expression patterns of several ROS-related DEGs in aged heart and skeletal muscle toward the patterns of young mice (Additional file 1: Fig. S12). Collectively, the above transcriptomic analyses reveal that hesperetin appears to delay aging and bring about a younger pattern of gene expression in a range of tissues of old mice, including cardiac muscle and skeletal muscle.

## Discussion

We have shown here that, in old mice, a Cisd2 activator such as hesperetin is able to act as an effective translational therapy for slowing down aging and promoting longevity via activation of Cisd2. Three findings are pinpointed by this study. Firstly, Cisd2 is able to be targeted pharmaceutically and activated by small compounds, one being hesperetin. Intriguingly, late-life treatment with hesperetin of old mice is able to increase Cisd2 expression to a level comparable to that of young mice. Leading from this, this enhanced level of Cisd2 expression appears to delay aging or even bring about the rejuvenation of aged tissues, thereby promoting longevity and extending a healthy lifespan. Secondly, hesperetin exerts many beneficial effects on various age-related structural defects and functional declines. Examples identified in old mice include hesperetin induced attenuation of age-related whole-body energy metabolic decline, a decrease in body fat accumulation, an amelioration of body lean loss, and an improvement in systemic glucose homeostasis. In addition, hesperetin also delays the aging of the cardiac and skeletal muscle, which is crucial to systemic metabolism. Most importantly, hesperetin functions mainly in a Cisd2-dependent manner and the therapeutic benefits of hesperetin disappear when Cisd2 gene is absent. Thirdly, oral administration of hesperetin during old age results in a younger transcriptome pattern that is similar to the pattern found in young mice and is distinctly different to that found in naturally aged mice. This beneficial or “youthful” profile brought about by hesperetin includes genes involved in metabolism, proteostasis, mitochondrial function and oxidative stress, cell death and senescence, as well as immune responses and inflammation. All of these pathways are associated with age-related biological processes.

### Hesperetin activates Cisd2 to extend healthy lifespan

In humans, several large meta-analyses of human populations had revealed that antioxidants, folic acid and B vitamins, as well as multivitamin and mineral supplements are ineffective in terms of increasing a healthy lifespan. There is no clear evidence in human studies to support the beneficial effect of these supplements in relation to all-cause mortality, cardiovascular disease, cancer, or cognitive function [58]. In animal studies using mammalian models, many studies have shown that treatment with antioxidant supplements, when aimed at extending lifespan, result in no overt effect or even can have a negative effect [59].

Remarkably, oral administration of hesperetin, when begun at a late-life stage, is able to extend healthy lifespan. Hesperetin is a flavanone aglycone found in citrus fruit peels. Previous studies revealed that hesperetin is converted into various conjugated metabolites after oral administration in rats and humans [60, 61]. Moreover, hesperetin is able to scavenge ROS and reduce lipid peroxidation without a cytotoxic effect [62]. Hesperetin is not only a strong peroxynitrite (ONOO^−^) scavenger, but also an effective inhibition of peroxynitrite-mediated nitration of tyrosine through electron donation [63]. In addition, hesperetin has cancer chemopreventive effects through a variety of targets related to oxidative and inflammatory processes [64]. Previously studies have also revealed that a variety of chemically induced cancers, such as urinary bladder, mammary, and colon cancers, are able to be inhibited by hesperetin based on cellular and animal models [65]. When metabolic disorders in animal models are considered, the following effects have been identified. In the heart, hesperetin in mice is able to inhibit pressure-overload cardiac remodeling [66], and attenuate post-infarction cardiac fibrosis through inhibition of the NF-kB pathway [67]. In the liver of hamsters, food supplemented with hesperetin and its chemical's metabolites, 3,4-dihydroxyphenylpropionic acid and 3-methoxy-4-hydroxycinnamic acid, would seem to be able to inhibit hepatic 3-hydroxy-3-methylglutaryl-coenzyme reductase and lowered plasma total cholesterol [68].

### Cisd2 activator hesperetin provides an alternative to CR mimetics

Calorie restriction (CR) with adequate nutrition is the only known non-genetic non-pharmacological intervention that results in a 20% to 40% increase in small mammals’ lifespan; it is also the most consistent method [69]. This approach delays or reduces the risk of many age-related diseases. The molecular signaling pathways mediating the anti-aging effect of CR include insulin/insulin growth factor-1, sirtuins, AMP-activated protein kinase, and the target of rapamycin, all of which form a complex interacting network. Several CR mimetics have been identified, including resveratrol (a SIRT1 activator), metformin (an AMP kinase activator) and rapamycin (an mTOR inhibitor). However, trials of these CR mimetics in healthy humans are currently limited [70]. Chronic rapamycin use can lead to hepatic gluconeogenesis, insulin resistance, severe glucose intolerance and even diabetes in small animals. Furthermore, 25% to 50% of patients treated with metformin suffer from poorly tolerated gastrointestinal side effects. Accordingly, an alternative regimen to the use of CR mimetics is needed as an intervention in geriatric medicine.

Here, we have successfully translated a genetic concept into a pharmaceutical approach in which a young-pattern of Cisd2 expression can be regained by oral administration of a Cisd2 activator, in this case, hesperetin, which is safe when used as a long-term supplement in food, even at the late-life stage of an aging mouse. Hesperetin is able to retard the aging process and ameliorate age-related functional declines based on this animal model.

## Conclusions

Hesperetin is the first compound we have tested as a proof-of-concept for the hypothesis that a Cisd2 activator will have an anti-aging effect. Our findings provide an experimental basis for using Cisd2 as a molecular target for the screening and development of novel compounds that are able to activate Cisd2 pharmaceutically with the goal of translating these drugs into clinical interventions that can be used in geriatric medicine. Most importantly, hesperetin can be rapidly delivered systematically to multiple organs and tissues in vivo. Additionally, it has no detectable in vivo toxicity after long-term oral administration for 6–7 months in mice, specifically when supplemented in food at a dose of 100 mg/kg/day, which has a human equivalent dose of 491 mg/60 kg/day. Accordingly, it will be of great interest to develop hesperetin as a medicinally or nutritionally functional food [68] for preventive purposes related to extending healthy lifespan and/or therapeutic purpose related to treating age-related diseases.

## Supplementary Information

**Additional file 1: Table S1.** The 60 noble herbs used to establish the compound library. **Table S2.** Diet composition of mouse food. **Fig. S1.** CISD2 reporter assay for hesperetin and its structural analogs. **Fig. S2.** Serum biochemical analyses revealed that hesperetin has no detectable in vivo toxicity after 6 months of treatment in the old WT mice. **Fig. S3.** Complete blood count (CBC) analyses revealed that hesperetin has no detectable toxicity on hematological parameters after 7 months of treatment in the old WT mice. **Fig. S4.** Compound concentrations of hesperetin, and two of the major conjugated metabolites, namely hesperetin-7-*O*-beta-d-glucuronide (H7G) and hesperetin-7-*O*-sulfate (H7S), in the dietary hesperetin-treated mice, as well as their bioactivity to enhance CISD2 expression in the HEK293-CISD2 reporter cells. **Fig. S5.** Comparison of whole body metabolic rate after hesperetin treatment for 1.5 and 6 months in old WT mice. **Fig. S6.** No significant difference in the glucose tolerance test (GTT) and insulin tolerance test (ITT) between the Veh-treated and Hes-treated old mice. **Fig. S7.** Hesperetin shifts the expression patterns of several insulin signaling-related differential expression genes (DEGs) in the aged livers toward the patterns of young mice. **Fig. S8.** Hesperetin delays skeletal muscle aging in a Cisd2-dependent manner. **Fig. S9.** Hesperetin delays cardiac aging in a Cisd2-dependent manner. **Fig. S10.** Subgroups of metabolism-related DEGs in the hearts and skeletal muscles. **Fig. S11.** Subgroups of proteostasis-related DEGs in the hearts and skeletal muscles. **Fig. S12.** Hesperetin decreases the levels of reactive oxygen species (ROS) and reactive nitrogen species (RNS), and shifts the expression patterns of several ROS-related DEGs in the aged hearts and skeletal muscles toward the patterns of young mice.

## Data Availability

The data that support the findings of this study are available from the corresponding author upon reasonable request.

## Abbreviations

Agl: Glycogen debranching enzyme
BAC: Bacterial artificial chromosome
CBC: Complete blood count
CR: Calorie restriction
DEG: Differentially expressed genes
ECG: Electrocardiography
EE: Energy expenditure
FDR: False discovery rate
G1P: Glucose 1-phosphate
G6P: Glucose 6-phosphate
G6pc: Glucose-6-phosphatase alpha
GO: Gene Ontology
GTT: Glucose tolerance tests
H7G: Hesperetin-7-*O*-beta-d-glucuronide
H7S: Hesperetin-7-*O*-sulfate
Hes: Hesperetin
HPLC: High performance liquid chromatography
ICD: Intercalated disc
ITT: Insulin tolerance tests
mcKO: Skeletal and cardiac muscles tissue-specific knockout
MGI: Mouse Genome Informatics
Pgm2: Phosphoglucomutase 2
PhK: Phosphorylase kinase
PKAα: Protein kinase A alpha
Pygl: Glycogen phosphorylase
ROS: Reactive oxygen species
RPKM: Reads per kilobase of exon model per million reads
TEM: Transmission electron microscopy
TG: Transgenic
VCO_2_: Carbon dioxide production rate
Veh: Vehicle
VO_2_: Oxygen consumption rate
WT: Wild-type
