# Supplementary material for: Hesperetin promotes longevity and delays aging via activation of Cisd2 in naturally aged mice
Source: J Biomed Sci. 2022 Jul 24;29:53. doi: 10.1186/s12929-022-00838-7 (PMC9310407; doi:10.1186/s12929-022-00838-7)
Supplement: Supplementary file 1 — Additional file 1: Table S1. The 60 noble herbs used to establish the compound library. Table S2. Diet composition of mouse food. Fig. S1. CISD2 reporter assay for hesperetin and its structural analogs. Fig. S2. Serum biochemical analyses revealed that hesperetin has no detectable in vivo toxicity after 6 months of treatment in the old WT mice. Fig. S3. Complete blood count (CBC) analyses revealed that hesperetin has no detectable toxicity on hematological parameters after 7 months of treatment in the old WT mice. Fig. S4. Compound concentrations of hesperetin, and two of the major conjugated metabolites, namely hesperetin-7-O-beta-d-glucuronide (H7G) and hesperetin-7-O-sulfate (H7S), in the dietary hesperetin-treated mice, as well as their bioactivity to enhance CISD2 expression in the HEK293-CISD2 reporter cells. Fig. S5. Comparison of whole body metabolic rate after hesperetin treatment for 1.5 and 6 months in old WT mice. Fig. S6. No significant difference in the glucose tolerance test (GTT) and insulin tolerance test (ITT) between the Veh-treated and Hes-treated old mice. Fig. S7. Hesperetin shifts the expression patterns of several insulin signaling-related differential expression genes (DEGs) in the aged livers toward the patterns of young mice. Fig. S8. Hesperetin delays skeletal muscle aging in a Cisd2-dependent manner. Fig. S9. Hesperetin delays cardiac aging in a Cisd2-dependent manner. Fig. S10. Subgroups of metabolism-related DEGs in the hearts and skeletal muscles. Fig. S11. Subgroups of proteostasis-related DEGs in the hearts and skeletal muscles. Fig. S12. Hesperetin decreases the levels of reactive oxygen species (ROS) and reactive nitrogen species (RNS), and shifts the expression patterns of several ROS-related DEGs in the aged hearts and skeletal muscles toward the patterns of young mice. [file 12929_2022_838_MOESM1_ESM.pdf]

Additional file 1

Supplementary Table S1 The 60 noble herbs used to establish the compound library.

Supplementary Table S2 Diet composition of mouse food.

Supplementary Fig. S1 CISD2 reporter assay for hesperetin and its structural analogs.

Supplementary Fig. S2 Serum biochemical analyses revealed that hesperetin has no detectable in vivo toxicity after 6 months of treatment in the old WT mice.

Supplementary Fig. S3 Complete blood count (CBC) analyses revealed that hesperetin has no detectable toxicity on hematological parameters after 7 months of treatment in the old WT mice.

Supplementary Fig. S4 Compound concentrations of hesperetin, and two of the major conjugated metabolites, namely hesperetin-7-O-beta-D-glucuronide (H7G) and hesperetin-7-O-sulfate (H7S), in the dietary hesperetin-treated mice, as well as their bioactivity to enhance CISD2 expression in the HEK293-CISD2 reporter cells.

Supplementary Fig. S5 Comparison of whole body metabolic rate after hesperetin treatment for 1.5 and 6 months in old WT mice.

Supplementary Fig. S6 No significant difference in the glucose tolerance test (GTT) and insulin tolerance test (ITT) between the Veh-treated and Hes-treated old mice.

Supplementary Fig. S7 Hesperetin shifts the expression patterns of several insulin signaling-related differential expression genes (DEGs) in the aged livers toward the patterns of young mice.

Supplementary Fig. S8 Hesperetin delays skeletal muscle aging in a Cisd2-dependent manner.

Supplementary Fig. S9 Hesperetin delays cardiac aging in a Cisd2-dependent manner.

Supplementary Fig. S10 Subgroups of metabolism-related DEGs in the hearts and skeletal muscles.

Supplementary Fig. S11 Subgroups of proteostasis-related DEGs in the hearts and skeletal muscles.

Supplementary Fig. S12 Hesperetin decreases the levels of reactive oxygen species (ROS) and reactive nitrogen species (RNS), and shifts the expression patterns of several ROS-related DEGs in the aged hearts and skeletal muscles toward the patterns of young mice.

Supplementary Table S1 The 60 noble herbs used to establish the compound library.

No	Species (Latin name)	Plant part used
1	*Acanthopanax gracilistylus* W. W. Smith	ACANTHOPANACIS CORTEX
2	*Achyranthes bidentata* Bl	ACHYRANTHIS BIDENTATAE RADIX
3	*Acorus tatarinoxjuii* Schott	ACORI TATARINOWII RHIZOMA
4	*Alisma plantago-aquatica* Linn.	ALISMATIS RHIZOMA
5	*Angelica pubescens* Maxim. f. biserrata Shan et Yuan	ANGELICAE PUBESCENTIS RADIX
6	*Artemisia scoparia* Waldst. et Kit.	ARTEMISIAE SCOPARIAE HERBA
7	*Asarum sieboldii* Miq.	ASARI RADIX ET RHIZOMA
8	*Asparagus cochinchinensis* (Lour.)Merr	ASPARAGI RADIX
9	*Atractylodes macrocephala* Koidz.	ATRACTYLODIS MACROCEPHALAE RHIZOMA
10	*Aucklandia lappa* Decne	AUCKLANDIAE RADIX
11	*Carpesium abrotanoides* L.	CARPESII FRUCTUS
12	*Cassia obtusifolia* L.	CASSIAE SEMEN
13	*Chrysanthemum morifolium* Ramat.	CHRYSANTHEMI FLOS
14	*Citrus reticulata* Blanco	CITRI EXOCARPIUM RUBRUM
15	*Clematis apiifolia* DC.	HERBA
16	*Cnidium monnieri* (L.) Cuss.	CNIDII FRUCTUS
17	*Coix lacryma-jobi* L. var. majuew (Roman.) Stapf	COICIS SEMEN
18	*Cucurbita moschata* Duch.	SEMEN
19	*Cuscuta chinensis* Lam.	CUSCUTAE SEMEN
20	*Cynanchum paniculatum* (Bunge) Kitagawa	CYNANCHI PANICULATI RADIX ET RHIZOMA
21	Dioscorea opposita Thunb	DIOSCOREAE RHIZOMA
22	*Dipsacus asper* Wall.ex Henry	DIPSACI RADIX
23	*Eucommia ulmoides* Oliv	EUCOMMIAE CORTEX
24	*Euryale ferox* Salisb	EURYALES SEMEN
25	*Gastrodia elata* Bl.	GASTRODIAE RHIZOMA
26	*Gentiana manshurica* Kitag	GENTIANAE RADIX ET RHIZOMA
27	*Glycyrrhiza uralensis* Fisch	GLYCYRRHIZAE RADIX ET RHIZOMA
28	*Juncus setchuensis* Buchen.	HERBA
29	*Kochia scoparia* (L.)Schrad.	KOCHIAE FRUCTUS
30	*Leonurus japonicus* Houtt	LEONURIFRUCTUS
31	*Ligustrum lucidum* Ait	LIGUSTRI LUCIDI FRUCTUS
32	*Lycium barbarum* L.	LYCII FRUCTUS
33	*Magnolia biondii* Pamp	MAGNOLIAE FLOS

34	*Malva verticillate* L.	MALVAE FRUCTUS
35	*Morinda officinalis* How	MORINDAE OFFICINALIS RADIX
36	*Nelumbo nucifera* Gaertn	NELUMBINIS RHIZOMATIS NODUS
37	*Ophiopogon japonicus* (L.f.)KerGawl.	OPHIOPOGONIS RADIX
38	*Peucedanum praeruptorum* Dunn	PEUCEDANI RADIX
39	*Plantago asiatica* L.	PLANTAGINIS SEMEN
40	*Platycladus orientalis* (L.) Franco	PLATYCLADI SEMEN
41	*Polygala tenuifolia* Willd	POLYGALAE RADIX
42	*Poria cocos* (Schw.) Wolf	PORIA
43	*Prinsepia uniflora* Batal	PRINSEPIAE NUX
44	*Rehmannia glutinosa* (Gaetn.) Libosch.	REHMANNIAE RADIX
45	*Rhaponticum uniflorum* (L.) DC.	RHAPONTICI RADIX
46	*Salvia miltiorrhiza* Bge.	SALVIAE MILTIORRHIZAE RADIX ET RHIZOMA
47	*Selaginella tamariscina* (Besiuv.) Spring	SELAGINELLAE HERBA
48	*Sesamum indicum* L.	SESAMI SEMEN NIGRUM
49	*Solanum lyratum* Thunb.	HERBA
50	*Sonchus oleraceus* L.	HERBA
51	*Sophora japonica* L.	SOPHORAE FRUCTUS
52	*Thlaspi arvense* L.	THLASPI HERBA
53	*Trachelospermum jasminoides* (Lindl.) Lem.	TRACHELOSPERMI CAULIS ET FOLIUM
54	*Tribulus terrestris* L.	TRIBULI FRUCTUS
55	*Typha angustifolia* L.	TYPHAE POLLEN
56	*Ulmus pumila* L.	CORTEX
57	*Vaccaria segetalis* (Neck.) Garcke	VACCARIAE SEMEN
58	*Vitex trifolia* L.	VITICIS FRUCTUS
59	*Ziziphus jujuba* Mill.	JUJUBAE FRUCTUS
60	*Ziziphus jujuba* Mill. var. spinosa (Bunge) Hu ex H. F. Chow	ZIZIPHI SPINOSAE SEMEN

Supplementary Table S2 Diet composition of mouse food.

AIN-93G Growth Purified Diet (TestDiet)		
**Ingredients**	**Sources (% in food)**	
Fat	• Soybean Oil (7.00)	
Carbohydrate	• Corn Starch (39.75)<br>• Dextrin (13.20)<br>• Sucrose (10.00)	
Protein	• Casein (20.00)<br>• L-Cystine (0.30)	
Minerals and Vitamins	• AIN 93G Mineral Mix (3.50)<br>• AIN 93 Vitamin Mix (1.00)	
Miscellaneous	• Powdered Cellulose (5.00)<br>• Choline Bitartrate (0.25)<br>• t-Butylhydroquinone (0.0015)	
**Energy (% kcal from)**		
Fat	16.17	
Carbohydrate	64.90	
Protein	18.93	
**Additives (% of reagent weight/food weight)**		
	**Vehicle diet**	**Hesperetin diet**
Propylene glycol	3.04	3.04
Hesperetin	-	0.07

Supplementary Fig. S1

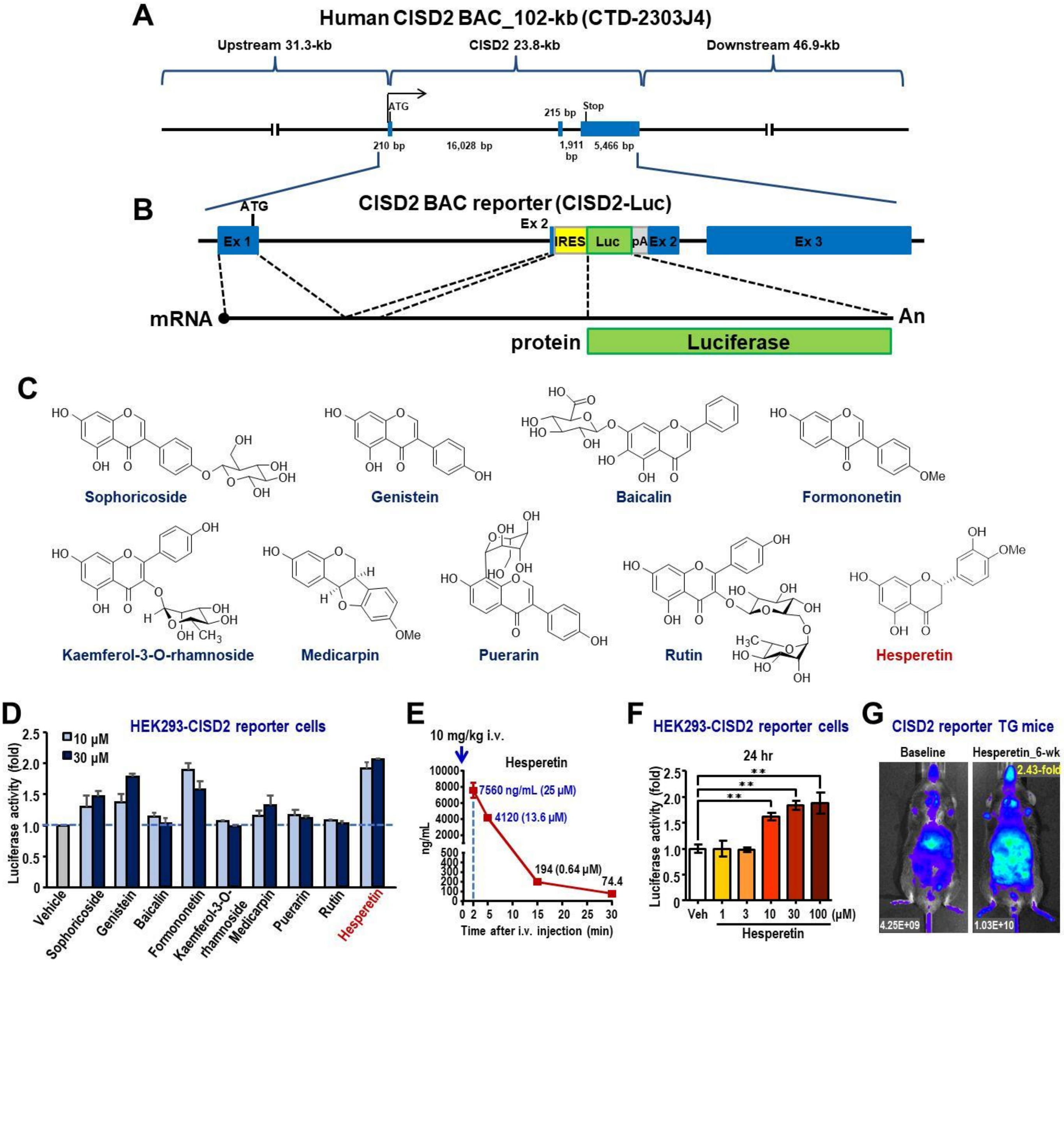

Supplementary Fig. S1 CISD2 reporter assay for hesperetin and its structural analogs. (A) The human BAC (bacterial artificial chromosome) clone CTD-2303J4, which contains the intact gene of human CISD2 in its native chromosomal setting, together with the flanking sequences of the upstream and downstream regions, was used to construct the CISD2 reporter gene. This BAC carries the entire genomic sequence of CISD2 coding region (23.8-kb), 31.3-kb upstream region and 46.9-kb downstream region. **(B)** The IRES-Luc-pA, namely an internal ribosome entry site (IRES), luciferase (Luc) and a polyA signal (pA), was inserted into the exon 2 of the CISD2 gene using in vivo recombineering-based method in E. coli. The IRES allows for the translation of luciferase protein in a cap-independent manner. This CISD2 BAC reporter was electroporated into HEK293 cells to established the HEK293-CISD2 reporter cells. In addition, it was also microinjected into fertilized eggs of C57BL/6 mice to generate transgenic (TG) mice, which will be used as the animal model for validating the potential CISD2 activator compounds. **(C)** Chemical structures of hesperetin and its structural analogs which have been analyzed for their activity to enhance CISD2 expression in the HEK293-CISD2 reporter cells. Sophoricoside and genistein from *Sophora japonica L.* (Supplementary Table S1) were initially identified as CISD2 activators. Seven structural analogs, including baicalin, formononetin, Kaemferol-3-O-rhamnoside, medicarpin, puerarin, rutin and hesperetin, were selected and examined for their activity to enhance CISD2 expression accordingly. **(D)** CISD2 reporter assay revealed that hesperetin, sophoricoside, genistein and formononetin can enhance CISD2 reporter, namely 1.5-2 fold of luciferase activity. HEK293-CISD2 reporter cells were treated with two different doses (10 and 30 μM) of the nine compounds as indicated. Luciferase activity was measured after compound treatment for 24 hours. Vehicle, 0.1% DMSO. **(E)** Compound concentrations of hesperetin in the serum of WT mice at different time points after intravenous (i.v.) injection of hesperetin (10 mg/kg). The molar concentrations of hesperetin are indicated in the brackets. **(F)** Hesperetin activates CISD2 reporter in HEK293 cells. The HEK293-CISD2 reporter cells were treated with different doses of hesperetin as indicated. Luciferase activity was measured after hesperetin treatment for 24 hours. Vehicle, 0.1% DMSO. In (D-F), all the experiments were performed and repeated for three independent times. Data are presented as mean ± SD. $^{*}p < 0.05$; $^{**}p < 0.005$ by one-way ANOVA with Bonferroni multiple comparison test. **(G)** IVIS analysis of in vivo luciferase activity was carried out using TG mice carrying the CISD2 BAC reporter before (baseline) and after dietary hesperetin treatment for 6 weeks (100 mg/kg/day provided in food).

Supplementary Fig. S2

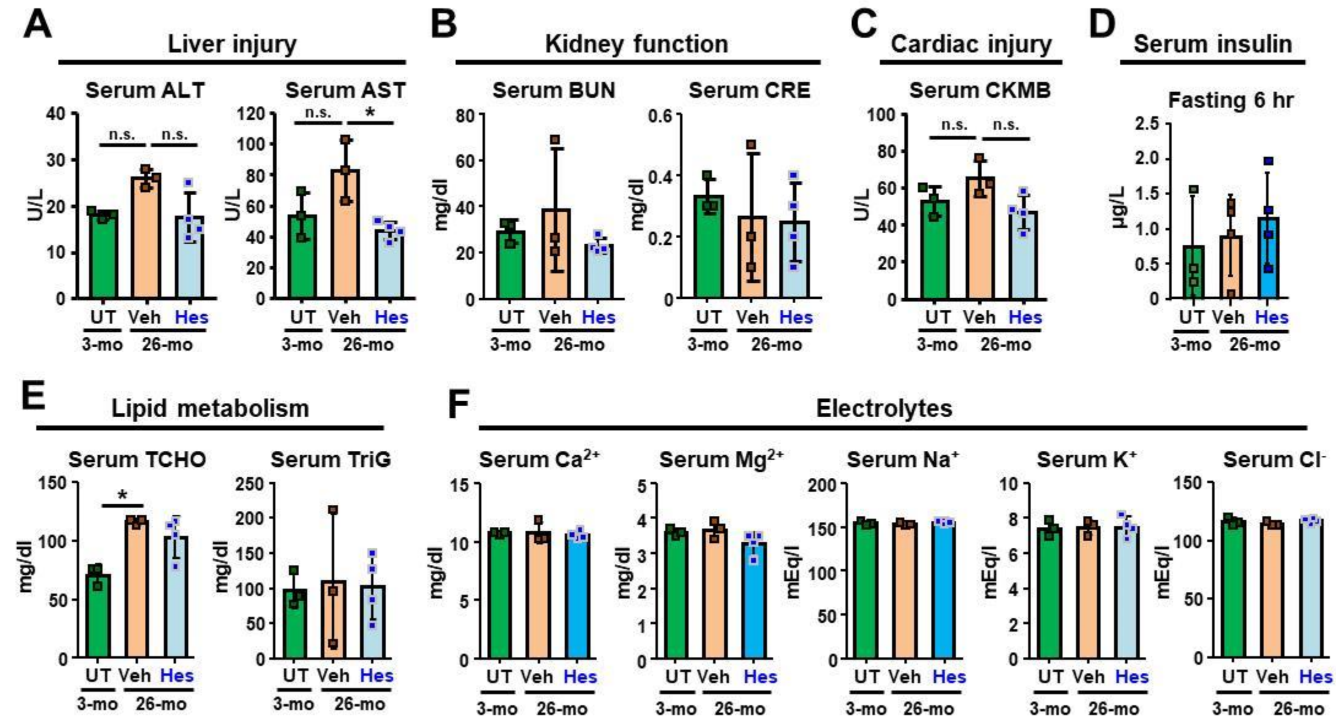

Supplementary Fig. S2 Serum biochemical analyses revealed that hesperetin has no detectable in vivo toxicity after 6 months of treatment in the old WT mice. (A) Liver damage markers (ALT and AST). **(B)** Kidney function (BUN) and kidney injury (CRE). **(C)** Cardiac injury (CKMB). **(D)** Serum insulin levels after 6 hours of fasting. **(E)** Serum TCHO and TriG levels. **(F)** Serum Ca^{2+}, Mg^{2+}, Na^{+}, K^{+}, and Cl^{-} levels. For the hesperetin treatment, 20-mo old WT mice were treated with dietary hesperetin (100 mg/kg/day provided in food) or vehicle control food (3.04% propylene glycol, w/w) for 6 months and sacrificed at 26-mo old. Data are presented as mean ± SD. *$p < 0.05$; **$p < 0.005$ by one-way ANOVA with Bonferroni multiple comparison test; not significant (n.s.). All the mice used in this study are males. Whole blood samples were collected from facial vein or cardiac puncture at sacrifice. The serum biochemical parameters were analyzed by Fuji Dri-Chem 4000i (FUJIFILM). Abbreviations: ALT, alanine aminotransferase; AST, aspartate aminotransferase; BUN, blood urea nitrogen; CRE, creatinine; CKMB, creatine kinase-myocardial band; TCHO, total cholesterol; TriG, triglycerides.

Supplementary Fig. S3

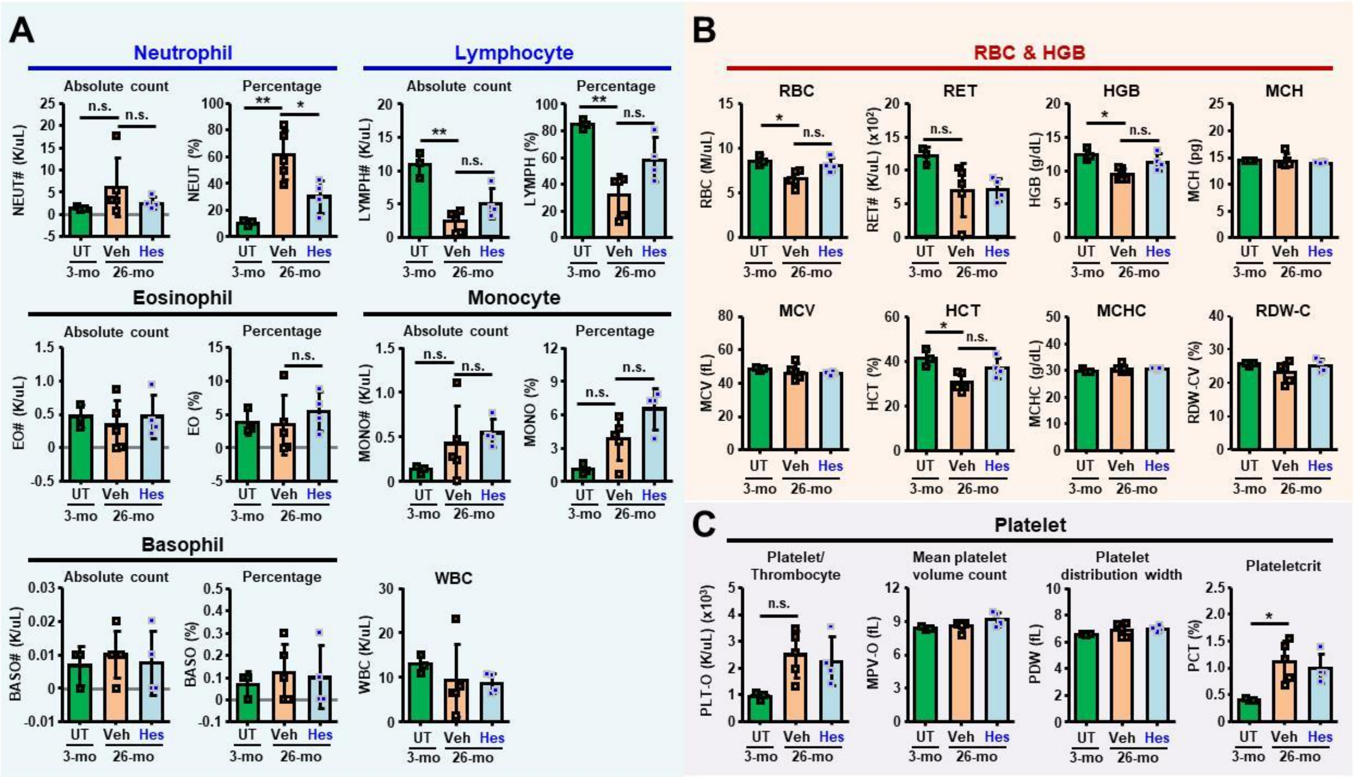

Supplementary Fig. S3 Complete blood count (CBC) analyses revealed that hesperetin has no detectable toxicity on hematological parameters after 7 months of treatment in the old WT mice. (A) White blood cells (WBC) parameters reveal that hesperetin (Hes) treatment appears to decrease the percentage of neutrophils compared with Veh-treated group of aged mice. **(B)** Parameters of red blood cells (RBC) and hemoglobin (HGB). **(C)** Parameters of platelets. For CBC analyses, 19-mo old WT mice were treated with dietary hesperetin (100 mg/kg/day provided in food) or vehicle control food (3.04% propylene glycol, w/w) for 7 months and collected whole blood samples at 26-mo old. Data are presented as mean ± SD. *p < 0.05; **p < 0.005 by one-way ANOVA with Bonferroni multiple comparison test; not significant (n.s.). All the mice used in this study are males. Whole blood samples were collected from facial vein using an EDTA (final concentration 5 mM) coated tube. The complete blood count was analyzed using a hematology analyzer (IDEXX ProCyte Dx). Abbreviations: HCT, hematocrit; HGB, hemoglobin concentration; MCH, mean corpuscular hemoglobin; MCHC, mean corpuscular hemoglobin concentration; MCV, mean corpuscular volume; RBC, red blood count; RDW, red cell distribution width; RET, reticulocytes count.

Supplementary Fig. S4

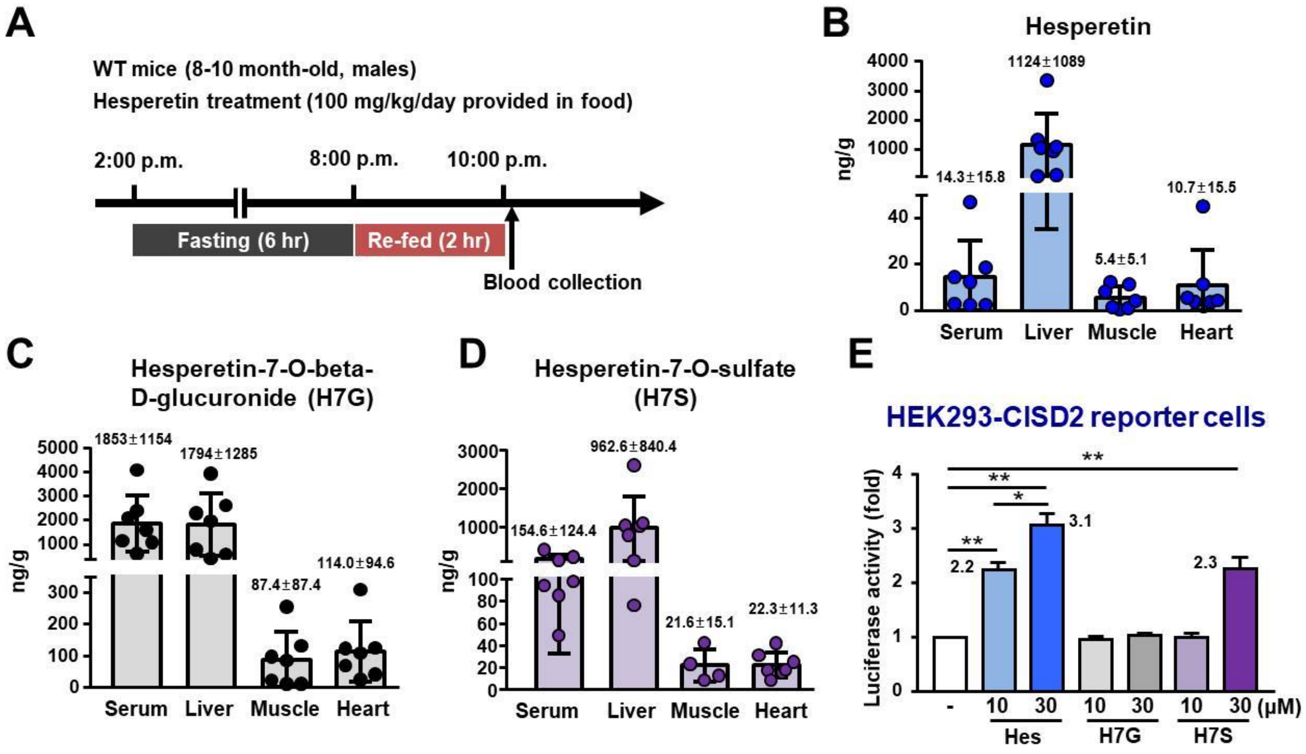

Supplementary Fig. S4 Compound concentrations of hesperetin, and two of the major conjugated metabolites, namely hesperetin-7-O-beta-D-glucuronide (H7G) and hesperetin-7-O-sulfate (H7S), in the dietary hesperetin-treated mice, as well as their bioactivity to enhance CISD2 expression in the HEK293-CISD2 reporter cells. (A) Protocol for determining the compound concentrations in the WT mice. Mice were fed the dietary hesperetin (100 mg/kg/day) ad libitum for 4 months until the day of sacrifice. To synchronize the food intake, the mice were fasted for 6 hours (2 p.m. to 8 p.m.) and re-fed the hesperetin food for 2 hours (8 p.m. to 10 p.m.). Compound concentrations of **(B)** hesperetin (Hes), **(C)** hesperetin-7-O-beta-D-glucuronide (H7G) and **(D)** hesperetin-7-O-sulfate (H7S) in the serum, liver, skeletal muscle (femoris) and heart of the dietary hesperetin-treated WT mice. **(E)** Hesperetin (10 μM) and a higher concentration (30 μM) of H7S activate the CISD2 reporter in the HEK293 cells. The HEK293-CISD2 reporter cells were treated with two different doses (10 and 30 μM) of hesperetin, H7G and H7S as indicated. Luciferase activity was measured after compound treatment for 24 hours. Vehicle, 0.1% DMSO. Data are presented as mean ± SD. $*p < 0.05$, $**p < 0.005$ by one-way ANOVA with Bonferroni multiple comparison test.

Supplementary Fig. S5

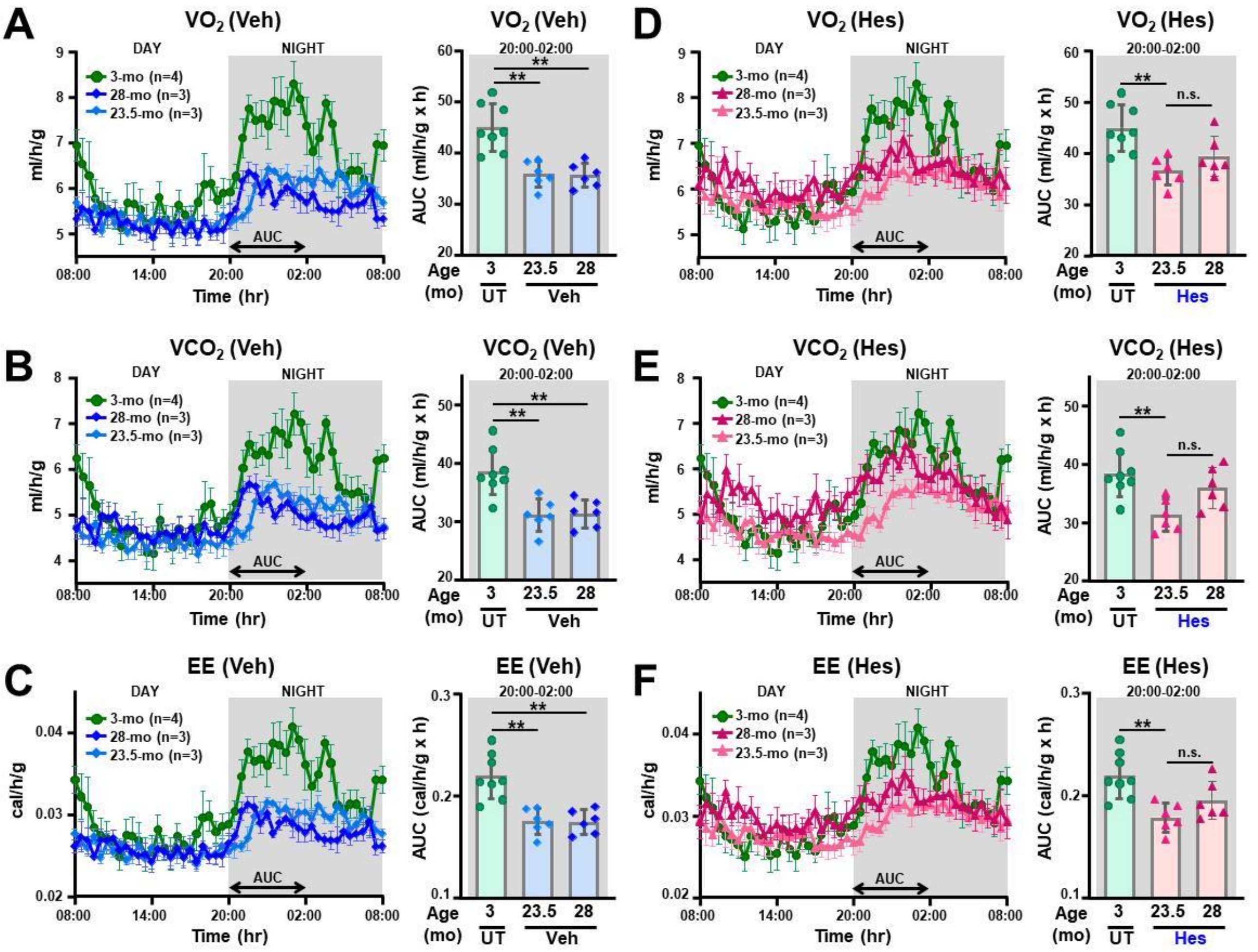

Supplementary Fig. S5 Comparison of whole body metabolic rate after hesperetin treatment for 1.5 and 6 months in old WT mice. (A-C) Whole body metabolic rate in the vehicle-treated old mice. Hour-to-hour average and quantification of **(A)** whole-body oxygen consumption (VO_2), **(B)** CO_2 production (VCO_2) and **(C)** energy expenditure (EE) during the light and dark periods after treatment with vehicle food for 1.5 months and 6 months. **(D-F)** Whole body metabolic rate in the hesperetin-treated old mice. Hour-to-hour average and quantification of **(D)** whole-body oxygen consumption (VO_2), **(E)** CO_2 production (VCO_2) and **(F)** energy expenditure (EE) during the light and dark periods after treatment with dietary hesperetin for 1.5 months and 6 months. The old mice (22-mo old) were treated with dietary hesperetin (100 mg/kg/day provided in food) or vehicle control food (3.04% propylene glycol, w/w). Their metabolic rate was monitored at 23.5-mo old (hesperetin or vehicle treatment for 1.5 months), and 28-mo old (hesperetin or vehicle treatment for 6 months). The metabolic rate of individual mouse was monitored for 48 hours. The area under curve (AUC) from 20:00 to 02:00 during dark period are quantified. For each mouse, two AUC quantitative values of each metabolic index calculated from the data of two cycles of 24-hr measurement are presented. Data for the VO_2, VCO_2 and EE are normalized to lean mass. Data are presented as mean ± SEM in the hour-to-hour metabolic monitoring. Data for quantification of AUC are presented as mean ± SD. *$p < 0.05$; **$p < 0.005$ by one-way ANOVA with Bonferroni correction for multiple comparison test; not significant (n.s.). All the mice used in this study are males. UT, un-treated.

Supplementary Fig. S6

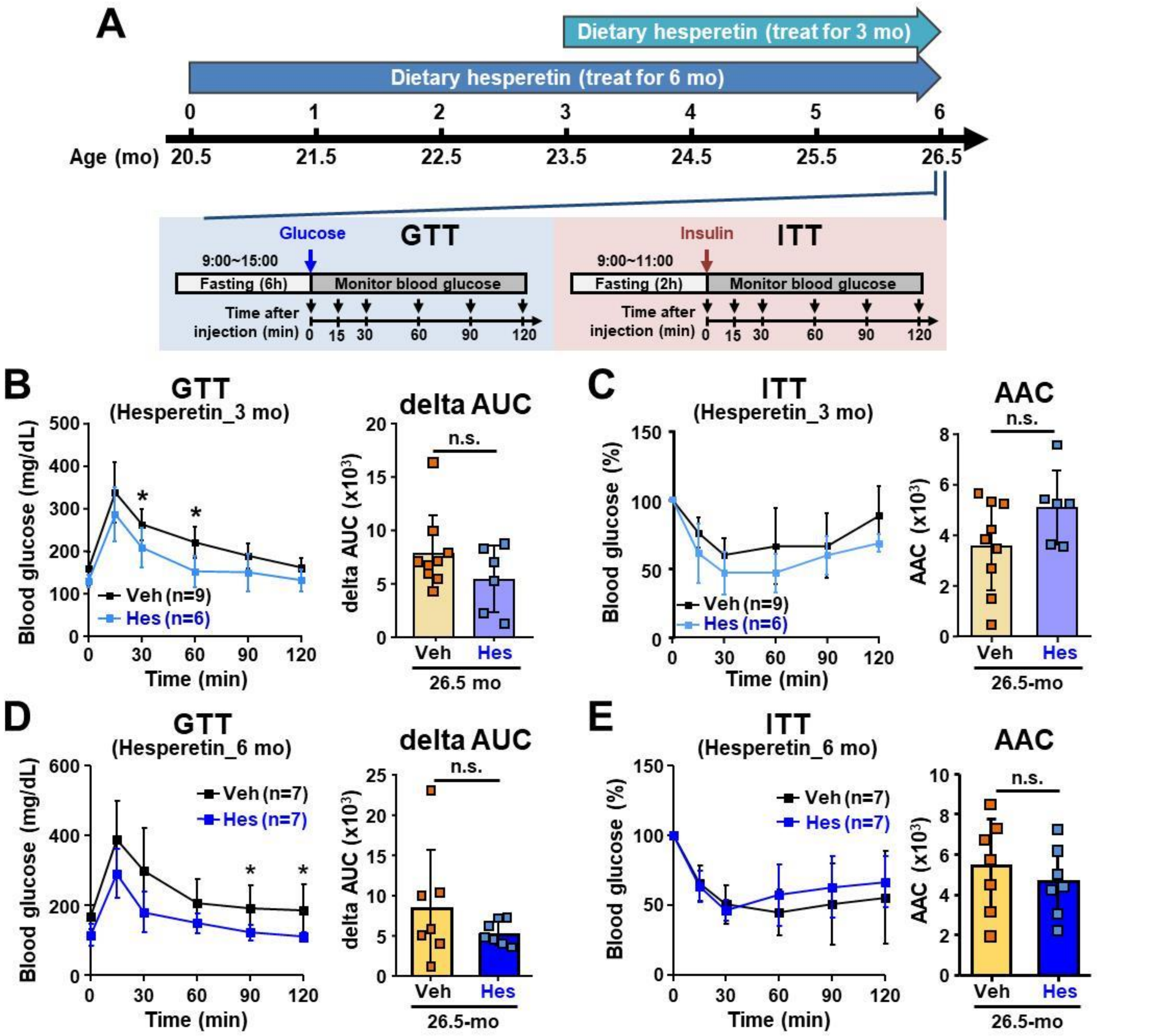

Supplementary Fig. S6 No significant difference in the glucose tolerance test (GTT) and insulin tolerance test (ITT) between the Veh-treated and Hes-treated old mice. (A) Protocols of GTT and ITT for the old mice treated with hesperetin for 3 months (from 23.5-mo to 26.5-mo) or 6 months (from 20.5-mo to 26.5-mo); GTT and ITT were carried out at 26.5-mo old. For the oral GTT, mice were orally administrated with glucose water (1.5 mg/g body weight) using a feeding needle after a 6 hours fasting (9 a.m. to 3 p.m.). For the ITT, mice were intraperitoneally injected with insulin (0.75 U/kg body weight) after a 2 hours fasting (9 a.m. to 11 a.m.). Blood samples were collected from tail vein before (0 min) and after the treatment at the indicated time points. **(B) (C)** Results of GTT and ITT for the mice treated with hesperetin or vehicle for 3 months. **(D) (E)** Results of GTT and ITT for the mice treated with hesperetin or vehicle for 6 months. For the GTT, quantification was measured by calculating the delta area under curve (delta AUC). For the ITT, quantification was measured by calculating the area above curve (AAC). Results are presented as mean ± SD. *$p < 0.05$; **$p < 0.005$ by Student's t test; not significant (n.s.). All the mice used in this study are males.

Supplementary Fig. S7

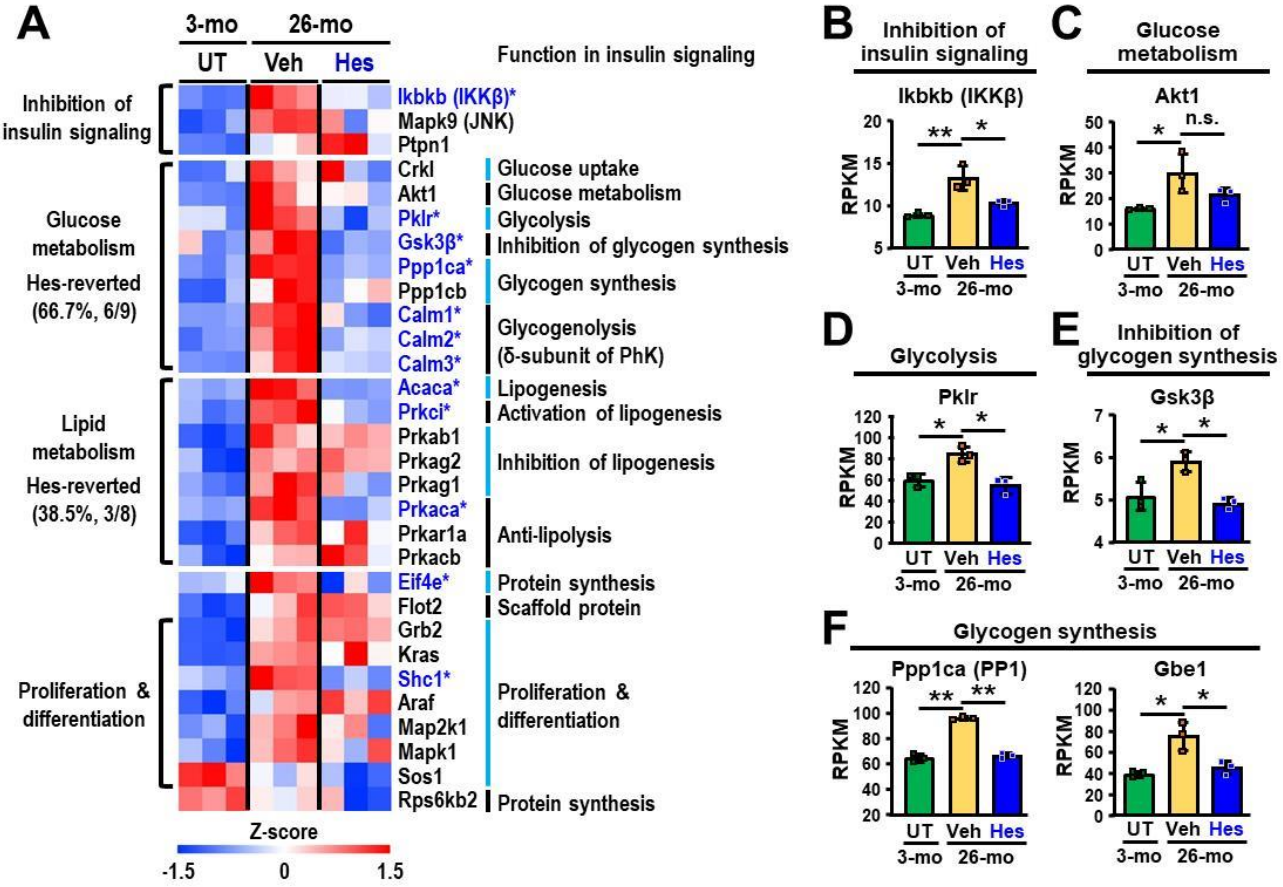

Supplementary Fig. S7 Hesperetin shifts the expression patterns of several insulin signaling-related differential expression genes (DEGs) in the aged livers toward the patterns of young mice. (A) Heatmap illustrating that hesperetin shifts the expression pattern of a panel of insulin signaling pathway-related DEGs, which are involved in glucose metabolism and lipid metabolism, in the aged livers toward the pattern of young livers; a total of 30 genes were shown (28 up-regulated and 2 down-regulated genes; 26-mo WT-Veh vs 3-mo WT, $p < 0.05$). The age-related differential expression genes that are significantly reverted by hesperetin are marked with an asterisk (26-mo WT-Hes vs 26-mo WT-Veh, $p < 0.05$). The gene list was referred from KEGG pathway (Insulin signaling pathway, mmu04910). **(B-F)** The mRNA levels of key enzymes involved in the pathways of inhibition of insulin signaling (B), glucose metabolism (C), glycolysis (D) and glycogen synthesis (E and F) in the livers of 3-mo WT, 26-mo Veh-treated and 26-mo Hes-treated WT mice (n = 3 mice per group) after hesperetin treatment for 5 months (from 21-mo to 26-mo). The mRNA levels were quantified by RNA-seq analysis. Results are presented as mean ± SD. *$p < 0.05$ by one-way ANOVA with Bonferroni multiple comparison test; not significant (n.s.).

Supplementary Fig. S8

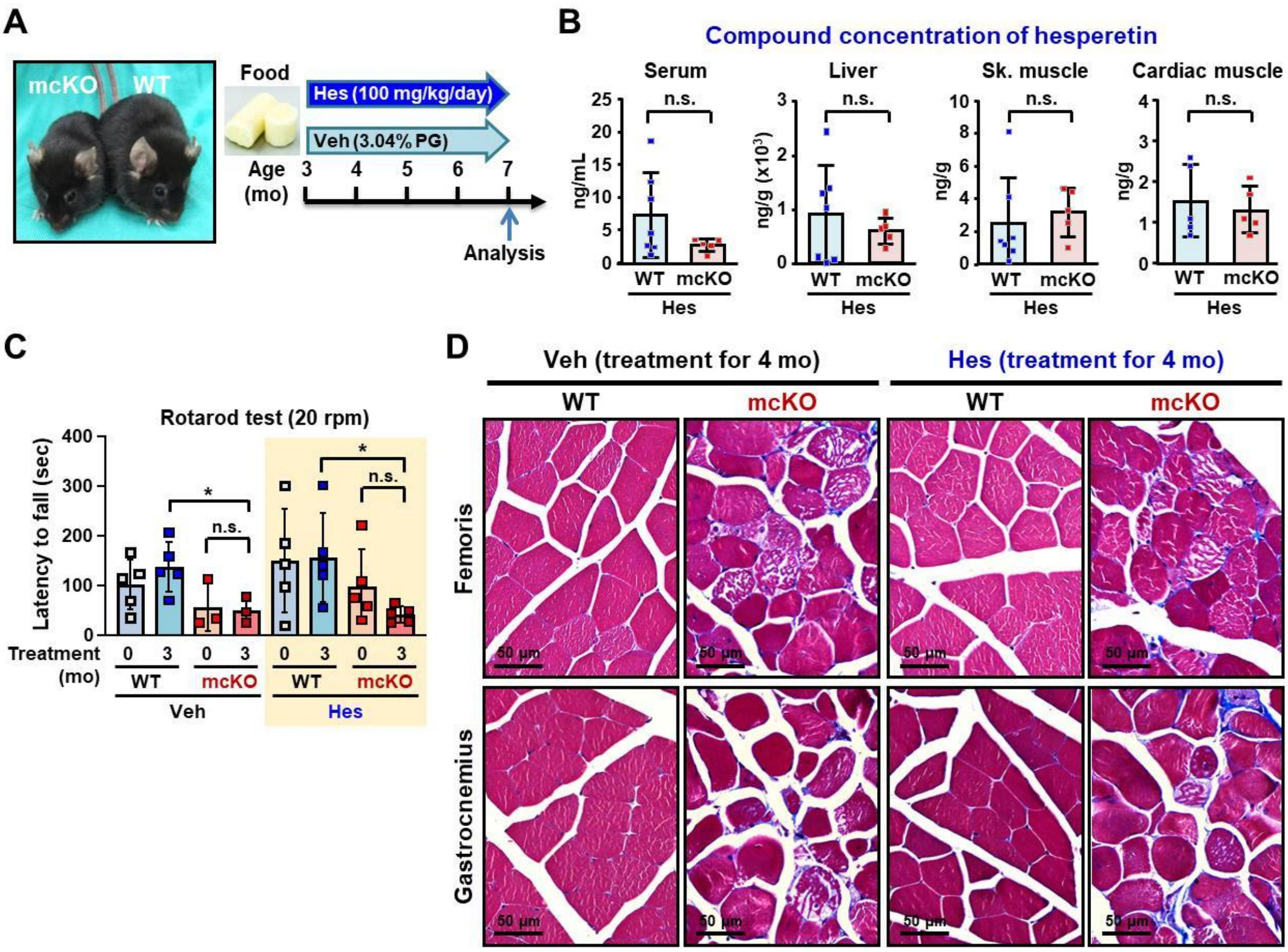

Supplementary Fig. S8 Hesperetin delays skeletal muscle aging in a Cisd2-dependent manner. (A) The 3-mo old WT and Cisd2 mcKO mice, which carry a Cisd2 KO background specifically in the skeletal and cardiac muscles, were treated with dietary hesperetin (100 mg/kg/day provided in food) or vehicle control food (3.04% propylene glycol, w/w) for 4 months and sacrificed at 7-mo old. **(B)** Compound concentration of hesperetin in the serum, liver, skeletal muscle (femoris) and cardiac muscle of WT and Cisd2 mcKO mice. **(C)** Rotarod test revealed that in the absence of Cisd2, hesperetin has no beneficial effect on the functional tests of skeletal muscle. **(D)** Histopathological analysis of skeletal muscles (femoris and gastrocnemius) revealed that in the absence of Cisd2, hesperetin has no beneficial effect on the structural damages and fibrosis. The paraffin-embedded muscle sections were examined by Masson's trichrome staining with the aim of detecting collagen. Data are presented as mean ± SD. *p < 0.05; **p < 0.005 by Student's t test; not significant (n.s.).

Supplementary Fig. S9

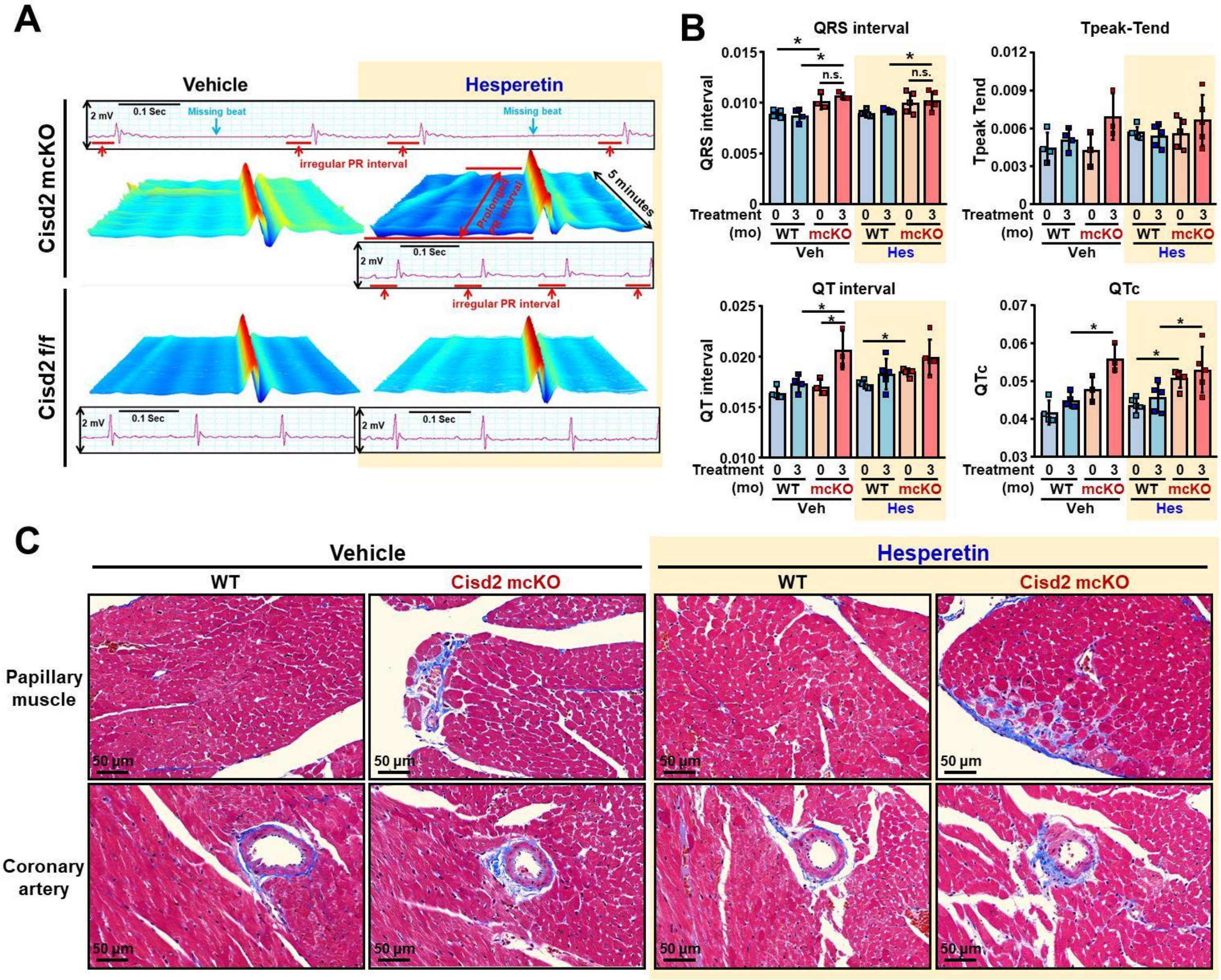

Supplementary Fig. S9 Hesperetin delays cardiac aging in a Cisd2-dependent manner. (A) Representative ECG tracings and continuous 5-minute waterfall plots recorded following anesthesia of the mice. Representative dysrhythmic ECGs, namely missing beat, prolonged and irregular PR interval, were found in the Cisd2 mcKO with or without hesperetin (Hes) treatment. **(B)** Quantification measurements obtained from 5-minute sequential beats of whole ECG tracings from baseline. **(C)** Histopathological analysis of cardiac muscle (left ventricle) revealed that in the absence of Cisd2, hesperetin has no beneficial effect on the structural injury and fibrosis. The paraffin-embedded myocardial sections (left ventricle) were examined by Masson's trichrome staining with the aim of detecting collagen. The 3-mo old WT and Cisd2 mcKO mice, which carry a Cisd2 KO background specifically in the skeletal and cardiac muscles, were treated with dietary hesperetin (100 mg/kg/day provided in food) or vehicle control food (3.04% propylene glycol, w/w) for 4 months and sacrificed at 7-mo old. ECG was monitored after hesperetin treatment for 3 months. In the absence of Cisd2, hesperetin has no beneficial effect on the ECG abnormalities, the histopathological injury and fibrosis in the hearts of Cisd2 mcKO mice. Quantification data are presented as mean ± SD and analyzed by two-way ANOVA with Bonferroni multiple comparison test. *$p<0.05$; not significant (n.s.).

Supplementary Fig. S10

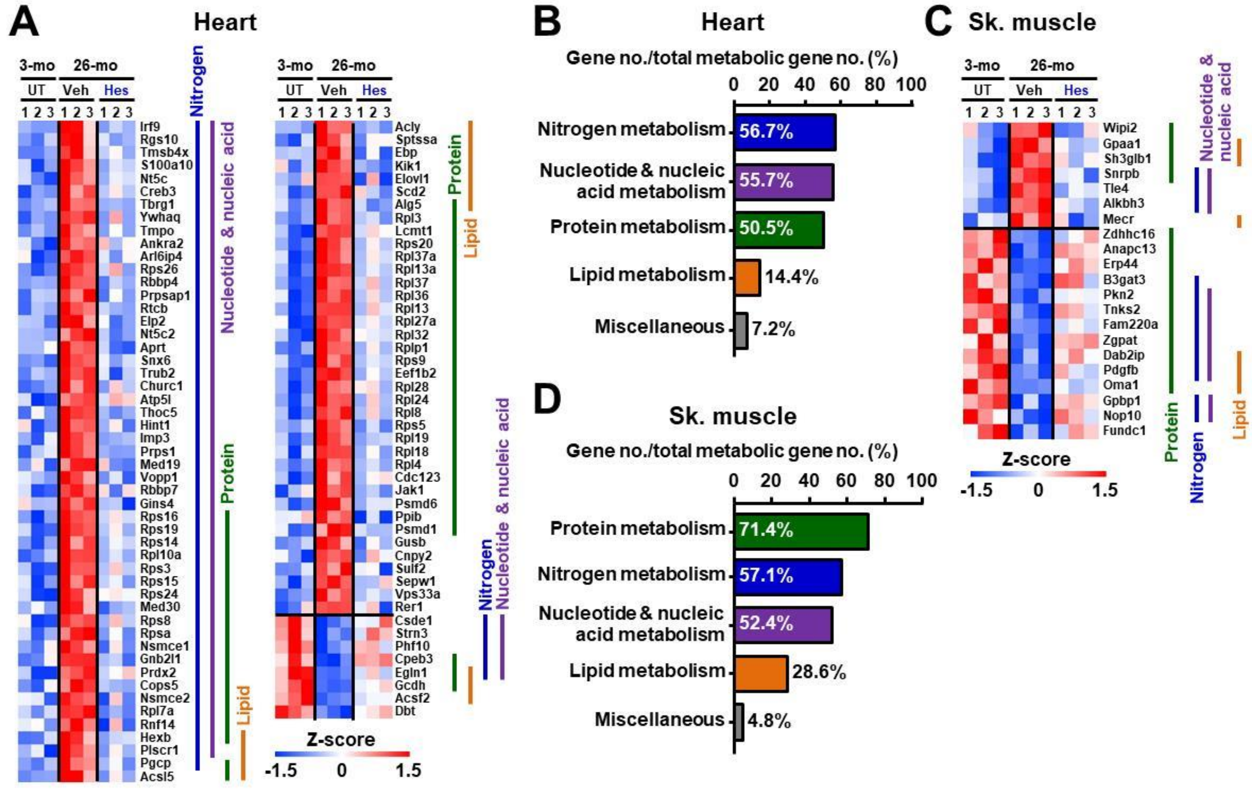

Supplementary Fig. S10 Subgroups of metabolism-related DEGs in the hearts and skeletal muscles. (A) Hesperetin shifts the expression pattern of a panel of metabolic DEGs, which are involved in nitrogen metabolism, nucleotide & nucleic acid metabolism, as well as protein and lipid metabolism, in the aged hearts toward the pattern of young hearts; a total of 97 DEGs were shown (89 up-regulated and 8 down-regulated genes). **(B)** Percentage of the metabolic DEGs in the aged hearts, which are reverted by hesperetin in panel (A), in different groups of metabolic pathways. **(C)** Hesperetin shifts the expression pattern of a panel of metabolic DEGs, which are involved in protein metabolism, nitrogen metabolism, nucleotide & nucleic acid metabolism and lipid metabolism, in the aged skeletal muscles (gastrocnemius) toward the pattern of young skeletal muscles; a total of 21 genes were shown (7 up-regulated and 14 down-regulated genes). **(D)** Percentage of the metabolic DEGs in the aged skeletal muscles, which are reverted by hesperetin in panel (C), in different groups of metabolic pathways. In this study, mice were treated with dietary hesperetin (100 mg/kg/day provided in food) or vehicle control food (3.04% propylene glycol, w/w) for 5 months (from 21-mo to 26-mo). The grouping of pathways was carried out by Mouse Genome Informatics (MGI) Gene Ontology term finder (pathway p-value < 0.05).

Supplementary Fig. S11

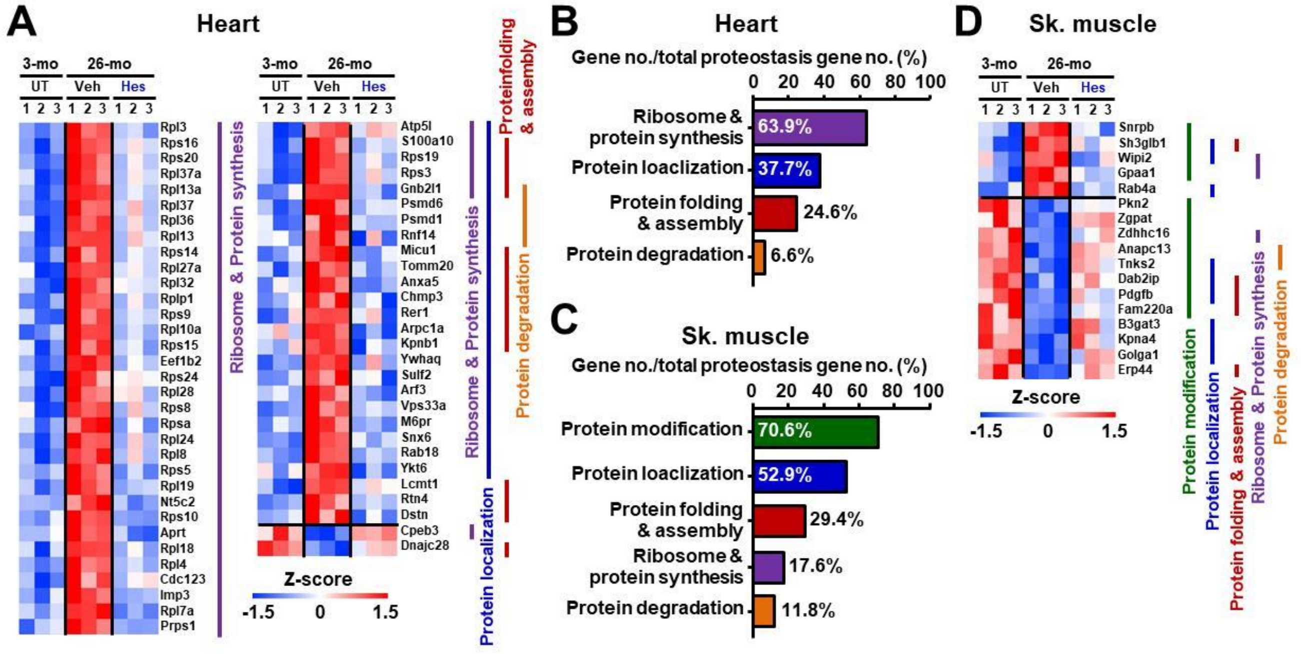

Supplementary Fig. S11 Subgroups of proteostasis-related DEGs in the hearts and skeletal muscles. (A) Hesperetin shifts the expression pattern of a panel of proteostasis-related genes, which are involved in ribosome & protein synthesis, protein folding & assembly, protein localization and protein degradation, in the aged hearts toward the pattern of young hearts; a total of 61 genes were shown (59 up-regulated and 2 down-regulated genes). **(B)** Percentage of the proteostasis-related DEGs in the aged hearts, which are reverted by hesperetin in panel A, in different groups of proteostasis-related pathways. **(C)** Hesperetin shifts the expression pattern of a panel of proteostasis-related genes, which are involved in protein modification, protein localization, ribosome & protein synthesis, protein degradation, as well as protein folding & assembly, in the aged skeletal muscle (gastrocnemius) toward the pattern of young skeletal muscle; a total of 17 genes were shown (5 up-regulated and 12 down-regulated genes). **(D)** Percentage of the proteostasis-related DEGs in the aged skeletal muscles, which are reverted by hesperetin in panel (C), in different groups of proteostasis-related pathways. In this study, mice were treated with dietary hesperetin (100 mg/kg/day provided in food) or vehicle control food (3.04% propylene glycol, w/w) for 5 months (from 21-mo to 26-mo). The grouping of pathways was carried out by Mouse Genome Informatics (MGI) Gene Ontology term finder (pathway p-value < 0.05).

Supplementary Fig. S12

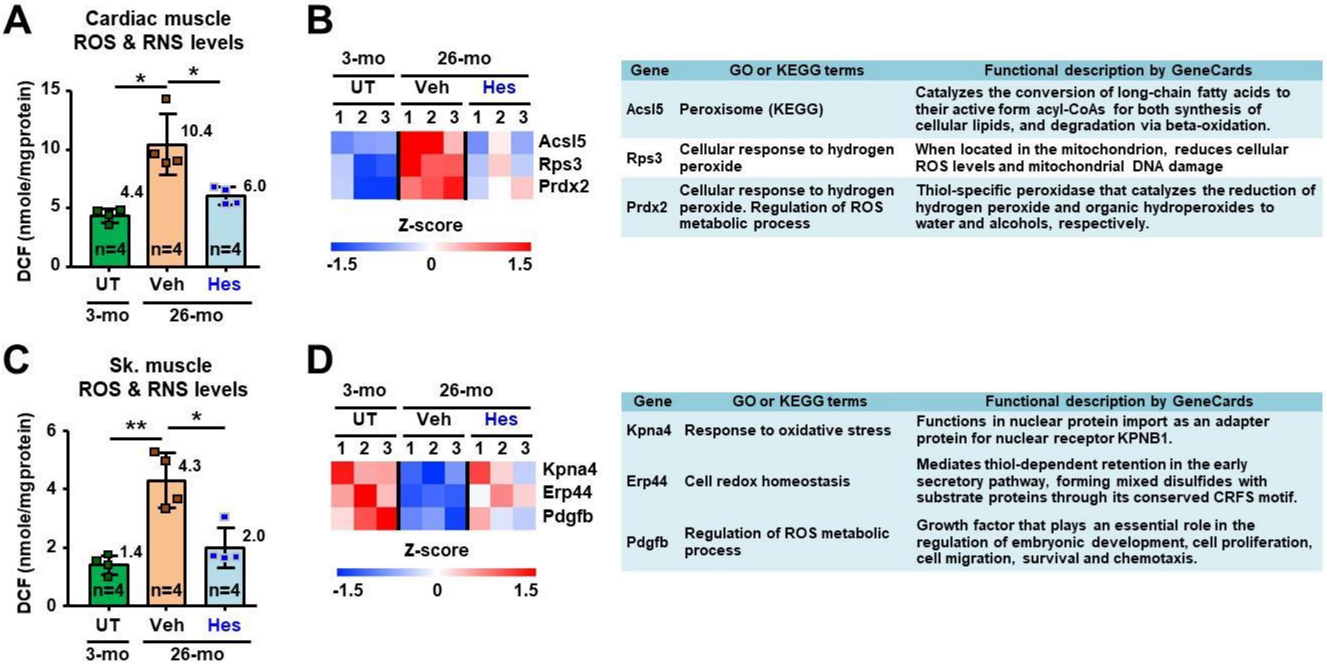

Supplementary Fig. S12 Hesperetin decreases the levels of reactive oxygen species (ROS) and reactive nitrogen species (RNS), and shifts the expression patterns of several ROS-related DEGs in the aged hearts and skeletal muscles toward the patterns of young mice. (A) Quantification of ROS and RNS levels in the heart by measuring DCF levels using cardiac muscle tissues. **(B)** Heatmap illustrating the ROS-related DEGs identified in the hearts of different groups of mice. Functional description by GeneCards is also provided for the DEGs identified. **(C)** Quantification of ROS and RNS levels in the skeletal muscles (gastrocnemius) by measuring DCF levels using skeletal muscle tissues. **(D)** Heatmap illustrating the ROS-related DEGs identified in the skeletal muscles (gastrocnemius) of different groups of mice. Functional description by GeneCards is also provided for the DEGs identified. In this study, mice were treated with dietary hesperetin (100 mg/kg/day provided in food) or vehicle control food (3.04% propylene glycol, w/w) for 5 months (from 21-mo to 26-mo). Results are presented as mean ± SD. $*p < 0.05$; $**p < 0.005$ by one-way ANOVA with Bonferroni multiple comparison test.
